# Formation of substituted dioxanes in the oxidation of gum arabic with periodate†

**DOI:** 10.1039/d2gc04923f

**Published:** 2023-04-26

**Authors:** Harmke S. Siebe, Andy S. Sardjan, Sarina C. Maßmann, Jitte Flapper, Keimpe J. van den Berg, Niek N. H. M. Eisink, Arno P. M. Kentgens, Ben L. Feringa, Akshay Kumar, Wesley R. Browne

**Affiliations:** a Stratingh Institute for Chemistry, Faculty of Science and Engineering, University of Groningen Nijenborgh 4 9747 AG Groningen The Netherlands w.r.browne@rug.nl; b Akzo Nobel Decorative Coatings BV Rijksstraatweg 31 Sassenheim 2171 AJ The Netherlands; c Akzo Nobel Car Refinishes BV Rijksstraatweg 31 Sassenheim 2171 AJ The Netherlands; d Magnetic Resonance Research Center, Institute for Molecules and Materials, Radboud University 6525 AJ Nijmegen The Netherlands a.kumar@science.ru.nl; e Dutch Polymer Institute (DPI) P.O. Box 902 5600 AX Eindhoven The Netherlands

## Abstract

Renewable polysaccharide feedstocks are of interest in bio-based food packaging, coatings and hydrogels. Their physical properties often need to be tuned by chemical modification, *e.g.* by oxidation using periodate, to introduce carboxylic acid, ketone or aldehyde functional groups. The reproducibility required for application on an industrial scale, however, is challenged by uncertainty about the composition of product mixtures obtained and of the precise structural changes that the reaction with periodate induces. Here, we show that despite the structural diversity of gum arabic, primarily rhamnose and arabinose subunits undergo oxidation, whereas (in-chain) galacturonic acids are unreactive towards periodate. Using model sugars, we show that periodate preferentially oxidises the *anti* 1,2-diols in the rhamnopyranoside monosaccharides present as terminal groups in the biopolymer. While formally oxidation of vicinal diols results in the formation of two aldehyde groups, only traces of aldehydes are observed in solution, with the main final products obtained being substituted dioxanes, both in solution and in the solid state. The substituted dioxanes form most likely by the intramolecular reaction of one aldehyde with a nearby hydroxyl group, followed by hydration of the remaining aldehyde to form a geminal diol. The absence of significant amounts of aldehyde functional groups in the modified polymer impacts crosslinking strategies currently attempted in the preparation of renewable polysaccharide-based materials.

## Abstract 2

Oxidation of the renewable polysaccharide feedstock gum arabic opens opportunities for derivatisation.

## Graphical abstract

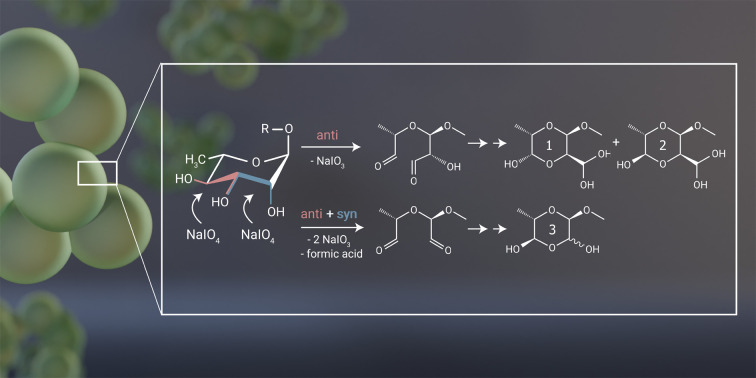

## 1. Introduction

The transition from oil-based to bio-sourced polymers is an essential step towards sustainability in materials science and technology. Biopolymer replacements typically require modification of their chemical structures, for example by oxidation, to obtain desired properties. Periodate (IO_4_^−^) is frequently employed to modify carbohydrate polymers, as it is soluble in water and is expected to provide aldehyde motifs through the oxidation of vicinal diols, which are amenable to chemical crosslinking.^2–8^ Importantly, despite it being a stoichiometric reagent, periodate can be regenerated using NaOCl or directly electrochemically from its iodate/iodine form,^9–12^ and although it is not competitive with catalysed oxidations using H_2_O_2_ and O_2_, the selectivity that can be achieved with periodate is still potentially useful.

The oxidation of diols with periodate was discovered by Malaprade in 1928.^13^ The current view on the mechanism of oxidation is that periodate coordinates to two vicinal diols, forming a cyclic intermediate prior to breaking the carbon–carbon bond and producing two aldehydes from the hydroxyl groups.^14^ This hypothesis is based on the observation that diols, which are locked in their *anti* configuration at an angle of 120° or greater, are not oxidised by periodate,^15^ whereas *anti* diols with smaller dihedral angles or *syn* diols are oxidised readily.^14^ Indeed, the expectation in literature is that the oxidation of a *syn* diol is favoured over that of an *anti* diol.^16^ Notably, for compounds that contain several neighbouring hydroxyl groups, the reaction is not limited to two of the hydroxyl groups, and double oxidation of a C–OH with two C–OH neighbours is manifested in the formation of formic acid.^14,17^

Beyond oxidising simple organic compounds,^18^ periodate has been applied specifically to oxidise carbohydrates, because of their high vicinal hydroxyl group content and the need for the reaction to be carried out under aqueous conditions, due to their limited solubility in organic solvents. After the oxidation, in aqueous solution the dialdehydes formed have been proposed to react with water, resulting in hydrates^14,19^ or hemiacetals.^20–23^ Recent examples of periodate modification of (complex) carbohydrates, such as alginate, cellulose, dextran and gum arabic, show its potential for use in bio-based food packaging,^24,25^ anti-bacterial coatings,^4^ and hydrogels for drug delivery.^26–30^

**Fig. 1:**
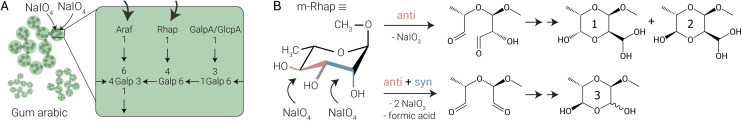
(A) The arabinogalactan main fraction of gum arabic obtained from *Acacia* s*enegal* var. s*enegal* (GA1), showing part of the monosaccharides present in the branched polymer chains, with the numbers indicating the respective C–OH position at which they are connected to neighbouring units.^1^ Common terminal monosaccharides are rhamnopyranoside (Rhap), arabinofuranoside (Araf) and the uronic acids galacturonic acid (GalpA) and glucuronic acid (GlcpA), which are connected to a branched galactopyranoside (Galp) backbone *via* their anomeric position. Sodium periodate (NaIO_4_) preferentially oxidises 1-Araf and 1-Rhap. (B) In the reaction of NaIO_4_ with the m-Rhap model sugar, mainly the *anti* diol is oxidised, forming 1 and 2. At higher equivalents of NaIO_4_, double oxidation occurs, in which both the *anti* and the *syn* diol are attacked, leading to the formation of 3 and formic acid. Products that would be formed solely from *syn* diol oxidation were not detected.

**Fig. 2:**
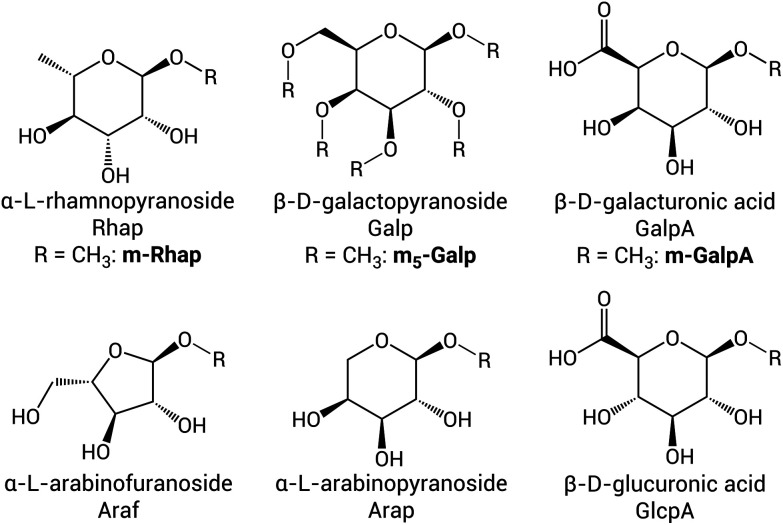
Monosaccharides present in GA1 listed with their full name, their abbreviations as used for the polymer (Fig. 1 and S1†) and for the model sugars studied here (if applicable) in bold. The connectivity of the monosaccharides in the polymer is indicated using numbers, which refer to the positions on the sugar ring, *e.g.* 1-Rhap is a terminal Rhap connected *via* its C1–OH, while 3-Araf-1 indicates an in-chain Araf unit, which is connected to its neighbours *via* its C3–OH and C1–OH.

Especially gum arabic is of interest, as it is soluble in water at high weight percentages, making it a promising ingredient for use in bio-based coatings. However, the feasibility of producing such coatings on an industrial scale depends, among other factors, on the reliability and continuity of the feedstock used and the reproducibility of its modification. As a bioproduct, the exact composition of gum arabic is subject to variation between species and local environmental factors. Gum arabic is obtained from the sap of either of two species of trees, *i.e. Acacia* s*enegal* var. s*enegal* (GA1) and *Acacia seyal* var. *seyal* (GA2), which differ in monosaccharide content, optical rotation, protein content and viscosity.^31,32^ Additionally, the elucidation of the gum arabic structure is not trivial, as it is a mixture of three complex polymers, of which the main part is an arabinogalactan that consists of a hyperbranched polysaccharide^33^ and a few percent of polypeptide.^34^ Nie *et al.* described the composition of the polysaccharide part of the main fraction of gum arabic and elucidated the connectivity of the rhamnopyranoside (Rhap), galactopyranoside (Galp), arabinofuranoside (Araf), arabinopyranoside (Arap), and uronic acid (galacturonic acid (GalpA) and glucuronic acid (GlcpA)) (Fig. 2) units using methylation and 2D NMR spectroscopy, for both GA1 ((Fig. 1A and Fig. S1†) and GA2.^1,32^ ATR-FTIR,^3,22,28,35–38^ NMR,^37^ and UV/Vis absorption^22^ spectra have been recorded of products of the periodate oxidation of gum arabic and the extent of the reaction followed using iodometric titrations.^23,29,39^ The amount of aldehyde formed in the product was quantified using hydroxylamine hydrochloride,^3,37,38,40–42^ hydroxyl ammonium chloride,^43,44^ and 2,4-dinitrophenylhydrazine^23,36,45^ titrations, or a TNBS assay.^46^ Removal of the iodate by-product is usually achieved by dialysis,^3,22,23,28,29,35,38,39,41–48^ or precipitation in ethanol^37^ or acetone^36^ and washing with water.

To date, a detailed analysis of the products obtained from the periodate oxidation of gum arabic has not been reported. Therefore, it is not known which parts of the polymer are oxidised by periodate and what the effect is of purification and isolation steps, such as extensive dialysis, precipitation and/or freeze-drying. Aldehyde formation has been assumed, but not validated in a rigorous manner spectroscopically.^3,22,23,28,29,35–38,41^

Here, we focus on the oxidation of GA1 using sodium periodate (NaIO_4_) (Fig. 1) and identify the selectivity of the oxidation by comparison with model sugars and using direct analysis with Raman, ATR-FTIR, solution-state ^1^H-NMR, ^13^C-NMR and 2D-NMR and solid-state cross-polarisation magic-angle spinning (CPMAS) ^13^C-NMR spectroscopy. We show that in the polymer, terminal rhamnopyranoside (1-Rhap) and arabinofuranoside (1-Araf) units are oxidised while (in-chain) arabinofuranoside (3-Araf-1) and galacturonic acid (1-GalpA and 4-GalpA-1) are unaffected. The oxidation of the rhamnopyranoside model sugar (m-Rhap) shows that, counter to expectations, the *anti* diol is oxidised rather than the *syn* diol. Notably, and contrary to earlier assignments,^3,36,37^ both in solution and the solid state, the major products observed do not contain aldehyde functional groups, but are ring-closed hydrated hemiacetals forming substituted dioxanes (Fig. 1B). The formation of stable hemiacetals impacts the effectiveness of strategies to form crosslinks based on aldehyde functionalisation.

## 2. Results and discussion

The application of modified gum arabic on a large scale requires the availability of consistent (reproducible) products, which is challenged by possible batch-to-batch variation in gum arabic itself and possible variation in the selectivity in the oxidation by periodate. In the present study, two batches of GA1 were oxidised several times with the same and different equivalents of NaIO_4_ to establish which part of the polymer is oxidised preferentially and what the reproducibility of the modification is. Further insight into the exact chemical modification of those parts was obtained by comparison with the oxidation of model sugars. These model sugars resemble the terminal monosaccharides present in gum arabic and are locked in their methyl acetal form to mimic the connection to a neighbouring monosaccharide in the polymer (Fig. 2). The oxidation of methyl-α-l-rhamnopyranoside (m-Rhap) and methyl-β-d-galacturonic acid (m-GalpA) was performed as model reaction for the oxidation of terminal units, as well as the oxidation of fully methylated β-d-galactopyranoside (m_5_-Galp), which is a model for the heavily substituted backbone sugar in the polymer (see Fig. S1† for the structure of GA1). The structural changes and reactivity found for the model sugars was used to interpret the changes observed in the spectra recorded of oxidised gum arabic (oxGA1), both in solution and in the solid state.

### 2.1. Reproducibility of the oxidation

**Fig. 3:**
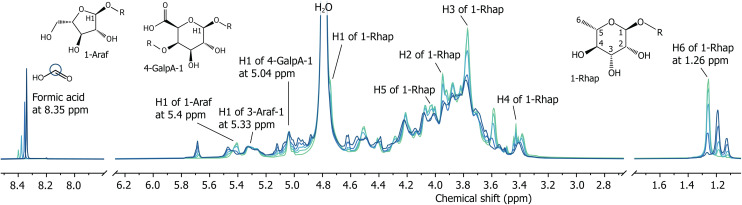
^1^H-NMR spectra of GA1 before (green) and after the addition of 0.05, 0.10, 0.20 and 0.30 eq. of NaIO_4_ (light to dark blue) in D_2_O, showing changes across the whole spectrum, among which the appearance of a formic acid signal at 8.35 ppm, a decrease in intensity of the C6 methyl group signal of rhamnopyranoside units (1-Rhap), and the appearance of two new C6 methyl groups of oxidised rhamnopyranoside at 1.18 and 1.12 ppm. The shift in the formic acid signal is due to a difference in formate/formic acid ratios present at different degrees of oxidation.

Raman spectroscopy was used to monitor the progress of the oxidation by following the consumption of periodate and the formation of iodate in the reaction mixture over time (Fig. S2–S4†). Consistently, full consumption of oxidant was observed within several minutes for both model sugars and gum arabic (see ESI† for details). Next to oxidant consumption, conversion of gum arabic was monitored directly using solution state ^1^H-NMR spectroscopy, by performing the oxidation in D_2_O and comparing spectra before and after the addition of periodate. The solution ^1^H-NMR spectrum of GA1 was assigned earlier by Nie *et al.*, and consists of many signals in the region between 3.2 and 5.5 ppm, among which the isolated signals of the H1 proton of 1-Araf at 5.4 ppm, H1 of in-chain 3-Araf-1 at 5.33 ppm and that of 4-GalpA-1 at 5.04 ppm. The signal of the methyl protons of rhamnopyranoside units is present at 1.26 ppm (Fig. 3).^1^^1^H-NMR spectra of different batches of GA1 are identical and match those reported in literature (Fig. S5†).^1^ Adding periodate to a solution of GA1 in D_2_O causes changes in intensities of some of the NMR signals present while leaving others unaffected (see Fig. 3 and S6, S7† for 2D NMR spectra showing the 1-Rhap signals in GA1). Importantly, also new product signals appear reproducibly in spectra of duplicate and triplicate measurements with essentially identical intensities in all of the reactions performed. The newly formed signals increase in intensity with increasing amounts of added periodate (Fig. S8–S11†). In contrast to the reproducibility of the NMR spectra, the FTIR spectra of the products are dependent on small variations in environment, such as pH (*vide infra*), and therefore vary considerably (Fig. S12†).

### 2.2. Selectivity of the oxidation

Comparing the solution-state ^1^H-NMR spectra of gum arabic before and after addition of periodate provides information about which of its units are oxidised. Addition of periodate to a solution of GA1 in D_2_O results in several signals appearing and disappearing, with only a few that do not overlap and can be used to determine the conversion of specific sugar units. For example, several signals for the rhamnopyranoside (1-Rhap) monosaccharide decrease in intensity, among which the methyl protons at 1.26 ppm, whilst two additional signals appear at 1.18 and 1.12 ppm (Fig. 3). Also the signal assigned to the H1 of terminal α-l-arabinofuranoside (1-Araf) at 5.4 ppm decreases, whereas the anomeric proton signals for in-chain α-l-arabinofuranoside (3-Araf-1) and β-d-galacturonic acid (4-GalpA-1) at 5.33 and 5.04 ppm stay unchanged. A consequence of the inertness of in-chain saccharides, however, is that the (reactive) terminal units are exposed to a higher effective periodate concentration, which can lead to multiple oxidation events at one monomer. Indeed, double oxidation occurs, as is evidenced from the formic acid (*vide supra*) signal present at 8.35 ppm in the ^1^H-NMR spectrum (Fig. 3).^14,49,50^

The selectivity for terminal sugar units such as 1-Rhap and 1-Araf over in-chain saccharides is expected, as they display (a larger number of) available hydroxyl groups, and the ends of the polymer chains may be more easily reachable by the oxidant. However, the observed selectivity is not solely attributable to the greater availability of terminal saccharides, but also to differences in chemical structure, as is apparent from a competition experiment using m-Rhap and m-GalpA. Rhap and GalpA are both present as terminal saccharides in the gum arabic polymer (Fig. 1). Both contain hydroxyl groups on their C2, C3 and C4 positions, and have two hydroxyl groups that are positioned *syn* and two that are *anti* with respect to each other (Fig. 2). The proton signals for GalpA, however, overlap with other signals in the ^1^H-NMR spectrum of (oxidised) gum arabic,^1^ and could not be monitored directly in the reaction of periodate with the polymer. Therefore, ^1^H-NMR studies on the model compound m-GalpA were performed. Mixing m-GalpA with periodate in D_2_O showed that oxidation occurs (Fig. S13†). However, oxidation proceeds solely when m-GalpA is the only substrate available; if periodate is added to a 1 : 1 mixture of m-GalpA and m-Rhap, only m-Rhap reacts (Fig. S14†). This indicates that not only the availability of hydroxyl groups is important in determining the selectivity of the periodate oxidation of GA1, but also differences in chemical reactivity of the diols of distinct monosaccharides play an important role in where periodate oxidises the polymer.

The integrated areas of the doublet of the C6 methyl group of the starting material and products can be used to calculate an approximate conversion of m-Rhap and terminal rhamnose (1-Rhap) units in GA1. It is apparent that for the oxidation of m-Rhap, the decrease in area of its C6 methyl signal matches well with the proportion of oxidant added, especially at lower NaIO_4_ equivalents (Fig. S15†). The amount of formic acid formed increases rapidly only above 1.0 eq. of NaIO_4_, with all starting material converted at around 1.2 eq. (Fig. S16†). In the case of gum arabic, the correlation is more complex, as there are multiple possible monosaccharide targets for the oxidation. Additionally, the amount of NaIO_4_ added is a number of equivalents with respect to the total number of monosaccharides in the polymer, and the effective amount of oxidant with respect to end group monosaccharides is significantly higher. This is supported by the observation that adding 0.05 eq. of NaIO_4_ leads to 19% instead of 5% conversion of Rhap in GA1.

#### 2.2.1. Elucidation of the product structure in solution

**Fig. 4:**
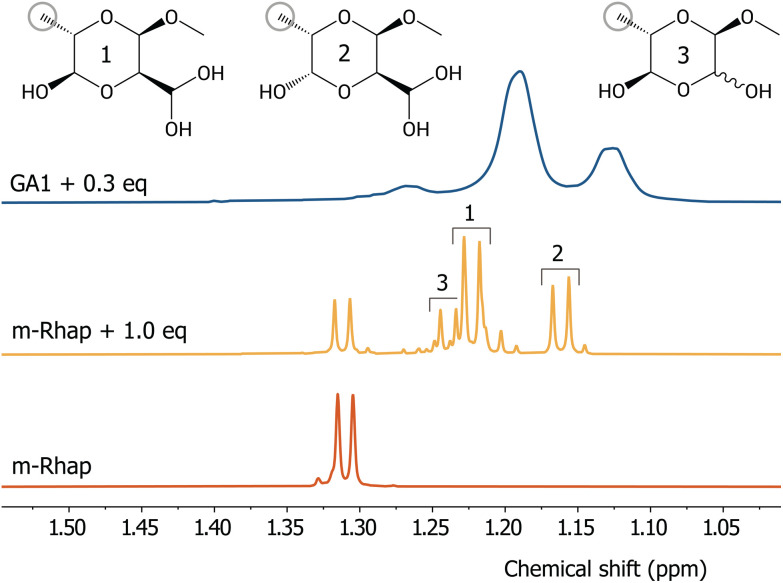
^1^H-NMR spectra (D_2_O, 600 MHz) of (red) m-Rhap, (yellow) m-Rhap oxidised with 1.0 eq. of NaIO_4_, and (blue) GA1 oxidised with 0.3 eq. of NaIO_4_. Several different distinct C6 methyl doublets are present in the spectrum of the model sugar, compared to broad bands observed in the spectrum of the polymer. The doublet at 1.31 ppm is remaining starting material and the doublets at 1.22, 1.16, and 1.24 ppm were assigned to product 1, 2, and 3, respectively (see ESI† for assignment using 2D NMR spectroscopy and comparison with computational spectra).

Formally, periodate oxidises diols to form dialdehydes. However, after oxidation, the ^1^H-NMR spectrum of GA1 only shows traces of aldehydes; the integrated area of the four small signals present between 9.3 and 9.7 ppm is 0.003 compared to the area of 3.00 for the rhamnopyranoside methyl protons (Fig. S17†). The fact that traces of aldehydes can be observed, precludes the absence of signals due to fast exchange and agrees with previously hypothesised hydration and (hemi)acetal formation,^49,51,52^ which is in turn consistent with the appearance of signals in the 5.4–5.8 ppm range (Fig. 3). Unfortunately, the complexity of the polymer and the broadening of the bands hinders thorough product analysis by 2D NMR spectroscopies. Therefore, the m-Rhap monosaccharide was used as a model compound to identify the products formed from the oxidation of the 1-Rhap units in GA1. Addition of NaIO_4_ to m-Rhap in D_2_O results in the appearance of two additional groups of signals for the C6 methyl protons in the ^1^H-NMR spectrum at a chemical shift similar to those observed in the oxidation of GA1, but with less line broadening (Fig. 4). Also other signals appear in the range of 3.4 to 5.8 ppm, with only traces of aldehydes observed at 9.5 ppm (integrated area of 0.04 compared to 1.0 for the C4 proton of product 1, Fig. S18†).

The main products formed upon oxidation of m-Rhap were assigned by 2D NMR spectroscopy as the ring-closed hydrated product of the single oxidation of the *anti* diol (product 1 and 2) and the ring-closed form of the doubly oxidised sugar (product 3). Their stereochemistry was determined using the difference in coupling constants between protons that are axial-equatorial (∼2 Hz) and axial-axial (∼8 Hz)^53^ (Fig. 4, see Fig. S19–S44† for the assigned (2D) NMR spectra of starting material and products and the possible mechanisms of the formation of the three products). Similar substituted dioxane structures to 1 and 2 were noted previously in the periodate oxidation of glucopyranosides^54^ and were proposed for other monosaccharides, such as rhamnopyranoside,^55^ as well. Product 3 was proposed to form with the C2 hydroxyl group oriented *syn* to the C1 O–Me substituent,^56^ but assigned as having the C2 hydroxyl group oriented *anti* later.^52^ Interestingly, product 1 and 2 are formed from the oxidation of the *anti* diol of m-Rhap, showing that periodate in this case does not prefer the oxidation of the *syn* diol, despite that it has been reported previously to be favourable for other saccharides.^57^ When adding 1.0 eq. of periodate, also double oxidation occurs, as evidenced by the formation of formic acid and product 3. Most likely this is due to the low amounts of m-Rhap left towards the end of the reaction, which makes double oxidation more likely than full conversion of the remaining starting material. Products formed solely from the oxidation of the m-Rhap *syn* diol were not identified.

Density Functional Theory (DFT) calculations of the various products that can form by oxidation of the *syn* and/or the *anti* diols of m-Rhap and subsequent ring-closing and hydration, show that the products 1 and 2 formed here, are the most thermodynamically favoured products, with the products that would result from the *syn* diol oxidation being 2.18–4.19 kcal mol^−1^ higher in energy. The four possible enantiomers of product 3 do not differ in energy significantly (see Table S2, and Fig. S45–S49 in the ESI† for details).

The band positions and shapes found in the solution ^1^H-NMR spectra of oxidised m-Rhap are similar to that of oxGA1, and although broadening precludes a direct correspondence of all signals, the absence of a significant aldehyde signal in either system, is a strong indication that also for oxGA1 in solution the aldehydes formed react with neighbouring hydroxyl groups and hydrate to form substituted dioxanes.

#### 2.2.2. Elucidation of the product structure in the solid state

**Fig. 5:**
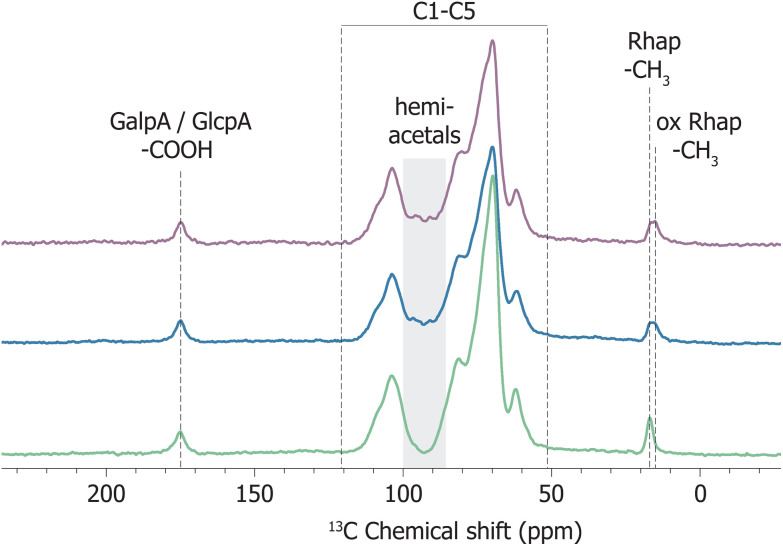
^13^C CPMAS spectra of (green) GA1, (blue) freezedried oxGA1 and (purple) oxGA1 precipitated in ethanol, using 0.2 eq. of NaIO_4_. Distinct signals are present for the methyl carbon of 1-Rhap at 17 ppm, with, after oxidation, an additional signal appearing at slightly lower chemical shift, as well as hemiacetal signals between 88 and 98 ppm. Signals indicative of aldehydes were not observed. All the spectra are normalised to the carbonyl carbons of GalpA and GlcpA at 175 ppm for comparison.

In solution, aldehyde functional groups react with neighbouring hydroxyl groups and hydrate by reacting with water. In the dry state, after the removal of water, in principle aldehydes can reform. Solid state NMR (ssNMR) spectroscopy was used to characterise oxGA1 and check for the presence of aldehydes in the dry state, which is of relevance for applications in materials. The solid state ^13^C CPMAS NMR spectra of GA1 and oxidised GA1 were recorded with a good signal to noise ratio (>25 for the carbonyl and methyl signals of the uronic acids and >100 for other sugar signals, Fig. 5).

In the spectrum of GA1, resonances at ∼17 ppm, 55–110 ppm and ∼175 ppm are assigned to the C6 methyl group of 1-Rhap, the C1 to C5 ring carbons, and the carbonyl carbon of uronic acids GlcpA and GalpA, respectively.^58^ After oxidation, the crude oxidised product was freezedried directly or precipitated in ethanol to assess the effect of the isolation method used on the product obtained. Notably, new signals due to aldehyde carbons, expected above 190 ppm, were not present in the ssNMR spectrum of either sample. Instead, signals with a chemical shift corresponding to hemiacetals are observed between 88 and 98 ppm, consistent with the products found for the solution state. The spectra of oxGA1 show a new rhamnopyranoside methyl carbon signal at lower chemical shift (see Fig. S50 and S51† for details), as is the case for the solution-state spectra (Fig. 4) and indicates oxidation of the Rhap units. The uronic acids signal at ∼175 ppm stays unchanged, which is in agreement with the lower reactivity of m-GalpA with respect to m-Rhap observed in solution and the absence of change for the signal of in-chain GalpA in the ^1^H-NMR spectra of oxGA1 (*vide supra*). This shows that also in the solid state, aldehydes are not present in significant amounts and the removal of water by either freeze-drying or precipitation in ethanol does not occur to an extent that aldehyde functional groups are reintroduced into oxGA1. Both isolation methods result in products with nearly identical spectra (Fig. S52†). An explanation for the absence of aldehydes after the removal of water, is that in oxGA1 instead of hydration of the not ring-closed aldehyde, hydroxyl groups from neighbouring sugar units may react intramolecularly with the aldehyde to form hemiacetals that are not affected by the removal of water.

**Fig. 6:**
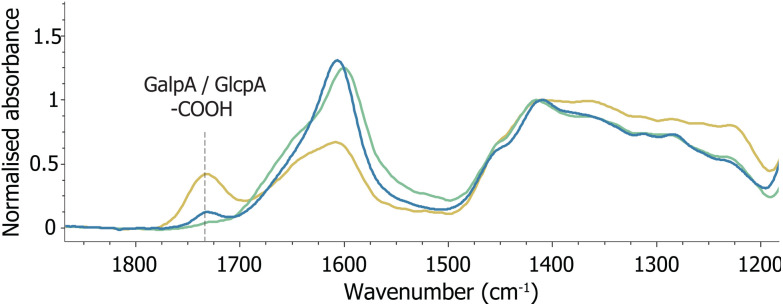
ATR-FTIR spectra of (green) GA1 and (blue) oxidised GA1 using 0.2 eq. of NaIO_4_, showing formation of a weak band at 1732 cm^−1^ assigned to protonated carboxylic acid C=O stretching and (yellow) acidified GA1 with HCl, showing the same band at 1732 cm^−1^. Spectra are baselined and normalised on the band at 1413 cm^−1^.

ATR-FTIR spectroscopy is often employed to characterise polymers oxidised with NaIO_4_. In the ATR-FTIR spectrum recorded of oxGA1, two additional bands were present, *i.e.* an iodate band at 780 cm^−1^ and a weak band at 1732 cm^−1^, assigned elsewhere to an aldehyde C=O stretch.^3,36,37^ The absorbance of the 1732 cm^−1^ band is low, which has been attributed to the possibility that a significant part of the aldehydes obtained undergo further reactions, both intra- and intermolecularly, to form hemiacetals and hemiketals.^20–23^ However, although the 1732 cm^−1^ band can be assigned to an aldehyde motif, the presence of carboxylates in the GA1 structure should be considered as well. The carboxylic acid COOH stretch appears upon protonation of the uronic acids GalpA and GlcpA present in gum arabic (Fig. 1 and 2). Acidification of a solution of GA1 with HCl, followed by lyophilisation, yields FTIR spectra with the same feature (Fig. 6 and S12†). Protonation of (oxidised) GA over the course of the reaction is plausible, due to formic acid formation by double oxidation of three vicinal diols (*vide supra*).

## 3. Conclusions

The use of sustainable polymers such as polysaccharides in new applications requires the modification of their chemical structure, for example by oxidation using periodate. The feasibility of using these modified polymers on an industrial scale depends, among others, on the reproducibility and exact nature of the modification. We show that the oxidation of gum arabic (GA1) is fast and reproducible. The ^1^H-NMR spectrum of oxidised GA1 (oxGA1) is complex, due to the presence of many overlapping signals of many different saccharides, but several distinct signals, such as those of the anomeric protons of rhamnopyranoside (Rhap), arabinofuranoside (Araf) and galacturonic acid (GalpA), show that terminal 1-Araf and 1-Rhap are targetted by NaIO_4_, leaving in-chain saccharides intact. Indeed, gum arabic is distinctly different from polysaccharides such as cellulose and starch, with a backbone that is highly substituted by polysaccharide side chains. Few to no “free” vicinal diols are available for oxidation by periodate and hence significant backbone depolymerisation is not expected. Competition experiments between model compounds m-Rhap and m-GalpA suggest that terminal uronic acids are not oxidised when other saccharides are also present. 2D NMR analysis of oxidised m-Rhap shows that products are formed which result from oxidation of the *anti* diol rather than the *syn* diol. This indicates that the two aldehydes formed are prone to intramolecular hemiacetal formation with the remaining C–OH present, resulting in substituted dioxanes.

Notably, DFT calculations show that the formed products 1 and 2 are the thermodynamically most stable, more so than the products that would be obtained by oxidation of the *syn* diol. Double oxidation of m-Rhap with excess (>1.0 eq.) periodate leads to the formation of formic acid and ring-closing to a different substituted dioxane (product 3). The band positions and shapes found in the solution ^1^H-NMR spectra of oxidised m-Rhap are similar to that of oxGA1, and although broadening precludes a direct correspondence of all signals, the absence of an aldehyde signal leads to believe that also for the dissolved polymer aldehydes ring-close and hydrate to form substituted dioxanes, either with hydroxyl groups in the same or surrounding sugar units.

Analysis of oxGA1 in the solid state confirms that aldehyde functional groups are not reformed upon removal of water. Solid state NMR spectroscopy shows no aldehyde carbons but signals corresponding to hemiacetals, for both oxGA1 isolated by precipitation in ethanol and by freeze-drying. Additionally, the band at 1732 cm^−1^ in the ATR-FTIR spectrum of oxGA1, which was previously assigned to an aldehyde C=O stretch, is also present in the spectrum of acidified GA1. Here we assign the 1732 cm^−1^ band as a C=O stretch of protonated carboxylic acids, due to protonation of the uronic acids present in the polymer upon double oxidation and the formation of formic acid.

The fact that the oxidation of gum arabic does not yield aldehydes but hemiacetals, influences greatly the effectiveness of follow-up modification steps, as usually aldehydes are envisioned to be used for crosslinking. Instead, the formed substituted dioxanes are relatively stable structures that first have to ring-open in order to react further. Exploring other pH ranges to shift the equilibrium from ring-closed products to aldehydes might be a feasible strategy. Alternatively, oxidised gum arabic could be used without additional crosslinkers, as oxidation alone resulted in a significant change in its physical properties, possibly due to crosslinking between polymer units and the addition of ions, which may already form a material that is sufficient for the intended applications.

## 4. Experimental

### 4.1 Materials

Gum arabic (GA1) (MW ∼250 000 g mol^−1^) was purchased from Acros Organics in two different batches (lot numbers A0398213 and A0413592). Dialysis tubing (Spectra/Por, M.W.C.O. 6–8 kDa) was obtained from Spectrum Laboratories Inc. CaCl_2_·2H_2_O was purchased from Acros Organics. m-Rhap was purchased from Carbosynth. Albumin from hen egg white was purchased from Fluka Biochemika. Sodium m-periodate, D_2_O, β-methyl-d-galactopyranoside, TEMPO, DIB, NaH, MeI, NH_4_Cl and MgSO_4_ were purchased from Sigma Aldrich. DMF, MeCN, MeOH, diethyl ether, ethyl acetate and pentane were purchased from Boom.

### 4.2 Synthesis of methylated monosaccharides

#### β-Methyl-d-galactopyranosiduronic acid (m-GalpA)

The procedure was adapted from the method described by Lu *et al.*^59^ β-Methyl-d-galactopyranoside (2.25 g, 11.6 mmol) was dissolved in MeCN (54 mL) and water (9 mL) and solid TEMPO (0.46 g, 2.9 mmol, 0.25 eq.) was added. The mixture was cooled with an ice-bath and (diacetoxyiodo)benzene (DIB, 9.33 g, 29 mmol, 2.5 eq.) was added slowly in portions. The mixture was stirred for 1 h at this temperature and subsequently stirred for another 20 h at r.t. whereupon the reaction was complete as monitored by TLC. The reaction was quenched with MeOH (125 μL, 2.9 mmol, 0.25 eq.). The solvent was removed by rotary evaporation until no methanol was left, and the remainder was precipitated in diethyl ether (10× the amount of the remaining volume). After filtration and drying of the solids *in vacuo* the product was obtained as white crystals. ^1^H-NMR (CD_3_OD) *δ*: 4.18 (2H, m); 3.94 (1H, s); 3.60 (3H, s); 3.55 (2H, m).

#### Methyl-2,3,4,6-tetra-*O*-methyl-β-d-galactopyranoside (m_5_-Galp)

To a flame dried Schlenk flask was added β-methyl-d-galactopyranoside (1.94 g, 10 mmol) in dry DMF (20 mL). The resulting solution was cooled to 0 °C (ice/water bath) and subsequently NaH (4 g, 100 mmol, 10 eq.) was added in portions. MeI (3.7 mL, 60 mmol, 6 eq.) was added over 1 h *via* a syringe pump. The resulting suspension was allowed to stir and warm up overnight before it was poured in saturated NH_4_Cl/ice mixture. The aqueous solution was extracted with diethyl ether (3 × 50 mL), the organic layers combined, dried over MgSO_4_, filtered and concentrated *in vacuo*. The crude solid was further purified *via* column chromatography (30% EtOAc in pentane) to give the desired product as a white crystalline powder (1.7 g, 6.8 mmol, 68%). Spectral data matched with those previously reported in literature.

### 4.3 Periodate oxidation of gum arabic

Typical conditions used for the synthetic scale oxidation of gum arabic involved dissolving 5 g of GA1 in 50 mL water (10 wt%) over 30 min, after which 0.20 eq. (1.25 g, 6.0 mmol) of NaIO_4_ was added to obtain 20% oxidised polysaccharide. The reaction was stirred magnetically for 10 min. The mixture was purified by dialysis against R.O. water over 3 days with five to six changes of water. Lyophilisation overnight using a CHRIST Alpha 2–4 LDplus freeze-dryer yielded a white foam which was characterised by ATR-FTIR spectroscopy (JASCO 7600 FTIR spectrometer).

### 4.4 Reaction monitoring with Raman spectroscopy

Reaction progress of the oxidation of gum arabic with sodium periodate was followed on a 1.5 mL scale using a Raman (*λ*_exc_ 785 nm) spectrometer comprising of a Avantes Raman probe connected *via* optical fibres to a ONDAX BT-785 nm laser source and Shamrock163i spectrograph equipped with a iDus420-OE CCD (Andor Technology) and 800 nm blazed 600 l mm^−1^ grating. Spectra were calibrated using cyclohexane as reference and processed with Spectragryph v.15. 750 μL of an aqueous sodium periodate solution (0.234 M, 0.2 eq.) was added to a quartz cuvette equipped with stirring bar, after which spectra were recorded continuously upon addition of 750 μL of a 20 w/v% solution of gum arabic. The periodate oxidation of model sugars was performed similarly, but using a 0.234 M monosaccharide solution instead of 20 w/v% gum arabic.

### 4.5 Product characterisation using solution-state NMR spectroscopy

The oxidation products were characterised directly by ^1^H-NMR spectroscopy by performing the reaction in D_2_O. The extent of oxidation as well as overoxidation was quantified by integration of the signal due to formic acid at 8.15 ppm, for different amounts of oxidant added. Several equivalents of solid NaIO_4_ (9.4, 19, 38 and 56 mg corresponding to 0.05, 0.10, 0.20 and 0.30 eq.) were added to gum arabic dissolved in D_2_O (10 wt%, 1.5 mL each) followed by vortexing to dissolve and react. ^1^H-NMR (600 MHz) spectra were recorded of the product mixtures directly using 64 scans. Reactions were performed in duplo. Model sugars were oxidised under the same conditions. All experiments were processed and analysed using MestReNova (Mnova) 14.2.

### 4.6 Product characterisation using solid-state NMR spectroscopy

All the experiments were performed using a Varian 3.2 mm HXY MAS probe used in ^1^H/^13^C double-resonance mode to achieve maximum sensitivity on a Bruker Neo 600 MHz (^1^H resonance) NMR spectrometer equipped with Bruker MAS-III unit. Each sample was packed into a thin wall 3.2 mm outer-diameter zirconia rotor. All the experiments were performed at room temperature and using a magic-angle spinning (MAS) frequency of 19.5 kHz to get sideband-free spectra. For ^1^H–^13^C CPMAS experiments, the CP contact time was set to 2.3 ms. A 70-to-100% ramp was used for the ^1^H CP spin-lock.^60,61^ Radio frequency (rf) field strengths were set to 100 kHz on the ^1^H and 50 kHz on the ^13^C channel. TPPM ^1^H decoupling of 100 kHz was used during acquisition. 8196, 8196, and 12 288 transients were accumulated for the ^1^H–^13^C CPMAS spectra of unoxidised sample, oxidised and freeze-dried sample, and oxidised and precipitated sample, respectively. The inter-scan delay and acquisition time were set to 1.7 s and 25 ms for all the samples. All the spectra were processed and analysed with Bruker ‘Topspin 4.1’ software. An exponential line broadening of 100 Hz was used while processing in all the spectra.

### 4.7 Product characterisation using FTIR spectroscopy

Fourier transform infrared (FTIR) spectra of freezedried product samples were recorded using a PerkinElmer Spectrum 400 FTIR spectrometer equipped with an ATR unit to probe the amount of aldehyde formed and to check for complete removal of the iodate by-product after precipitation or dialysis. ATR-FTIR spectra of acidified gum arabic were recorded by dissolving GA1 in 1.0 mM HCl (0.4 g in 2.0 mL, 20 w/v%) and adding droplets of 1 M HCl until pH 3 was reached, after which the solution was freeze-dried.

## Conflicts of interest

There are no conflicts to declare.

## Supplementary Material

GC-025-D2GC04923F-s001
