# Supplementary material for: Formation of substituted dioxanes in the oxidation of gum arabic with periodate
Source: Green Chem. 2023 Apr 26;25(10):4058–66. doi: 10.1039/d2gc04923f (PMC10202368; doi:10.1039/d2gc04923f)
Supplement: GC-025-D2GC04923F-s001 [file GC-025-D2GC04923F-s001.pdf]

Electronic Supplementary Material (ESI) for Green Chemistry.
This journal is © The Royal Society of Chemistry 2023

Supporting Information

Formation of substituted dioxanes in the oxidation of gum arabic with periodate

Harmke S. Siebe,[a] Andy S. Sardjan,[a] Sarina C. Maßmann,[a] Jitte Flapper,[b] Keimpe J. van den Berg,[c] Niek N. H. M. Eisink,[a] Arno P. M. Kentgens,[d] Ben L. Feringa,[a] Akshay Kumar,*[d,e] and Wesley R. Browne*[a]

Contents

1 The structure of gum arabic *Acacia senegal* var. *senegal* (GA1) 2

2 Reaction monitoring with Raman spectroscopy 2

3 Batch variation in GA1 4

4 2D NMR spectra of GA1 5

5 Reproducibility of the oxidation of GA1 7

6 The oxidation of m-GalpA 10

7 Competition experiment m-Rhap and m-GalpA 11

8 Oxidation of m-Rhap 12

9 Traces of aldehyde 13

10 Oxidation of m-Rhap 14

11 DFT calculations 35

12 Solid state ^{13}C NMR spectroscopy 41

[a] *Stratingh Institute for Chemistry, Faculty of Science and Engineering, University of Groningen, Nijenborgh 4, 9747 AG Groningen, The Netherlands. E-mail: w.r.browne@rug.nl*

[b] *Akzo Nobel Decorative Coatings BV, Rijksstraatweg 31, Sassenheim, 2171 AJ, The Netherlands.*

[c] *Akzo Nobel Car Refinishes BV, Rijksstraatweg 31, Sassenheim, 2171 AJ, The Netherlands.*

[d] *Magnetic Resonance Research Center, Institute for Molecules and Materials, Radboud University, 6525 AJ Nijmegen, The Netherlands. Email: a.kumar@science.ru.nl*

[e] *Dutch Polymer Institute (DPI), P.O. Box 902, 5600 AX Eindhoven, The Netherlands*

1. The structure of gum arabic *Acacia senegal* var. *senegal* (GA1)

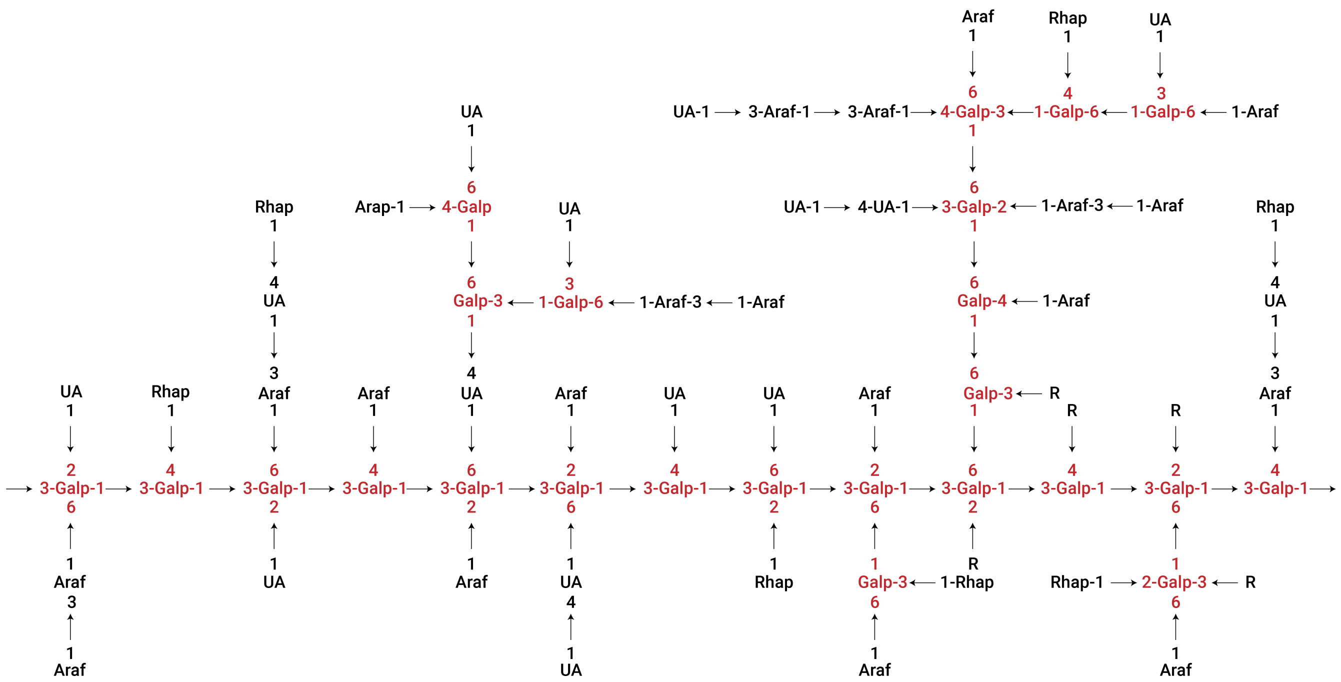

Figure S1: Proposed structure of GA1, with as consitutent monosaccharides Rhap = α-L-rhamnopyranose, UA = galacturonic (GalpA) or glucuronic acid (GlcpA) in the β-D-pyranose form, Arap = α-L-arabinopyranose, Araf = α-L-arabinofuranose and in the backbone (in red) Galp = β-D-galactopyranose. Numbers indicate which carbon of the monosaccharides are attached to which neighbouring ones via a glycosidic linkage. Adapted with permission from Nie et al.[1] Copyright © 2012 Published by Elsevier Ltd.

2. Reaction monitoring with Raman spectroscopy

The reaction times reported for the oxidation by periodate are up to hours,[2–5] and are reported to depend on temperature and pH,[6] the presence of metal salts,[7] and the concentration of periodate.[4,8,9] As reaction times reported for the periodate oxidations of 1,2-diols vary substantially, in the present study in-line monitoring of the conversion of periodate to iodate was carried out using Raman spectroscopy as described earlier.[10] The Raman spectrum of periodate (IO_4^-) shows two bands at 789 and 851 cm^{-1}. Its reduction product, iodate (IO_3^-), has a characteristic broad Raman band at 798 cm^{-1} (Fig. S2-A). As gum arabic has a low Raman scattering cross-section with a broad distribution of functional group types, significant changes in its Raman spectrum are not apparent over the course of the reaction. Several of the model sugars show more distinct bands, but for comparison, all rates were determined by adding solutions of (poly)saccharide to solutions of periodate under rapid stirring and following solely the intensity of the periodate bands over time.

Gum arabic (GA1)

The oxidation of gum arabic with periodate, followed by Raman spectroscopy, shows full conversion of IO_4^- to IO_3^- within 3.5 min (Fig. S2-B).

Model sugars

Compared to the polymer, the oxidation of model sugars proceeds on a similar timescale and not over several hours as reported elsewhere for mono- and dissaccharides.[4] Nevertheless, the reaction of periodate with m-Rhap takes 300 s (Fig. S2-C), in contrast to the oxidation of GA1, which proceeds mostly during the initial mixing time. Importantly, the addition of protected m_5-Galp does not result in reduction of IO_4^-, indicating also that loss of periodate due to light or heating from the laser can be disregarded (Fig. S2-D and Fig. S3).

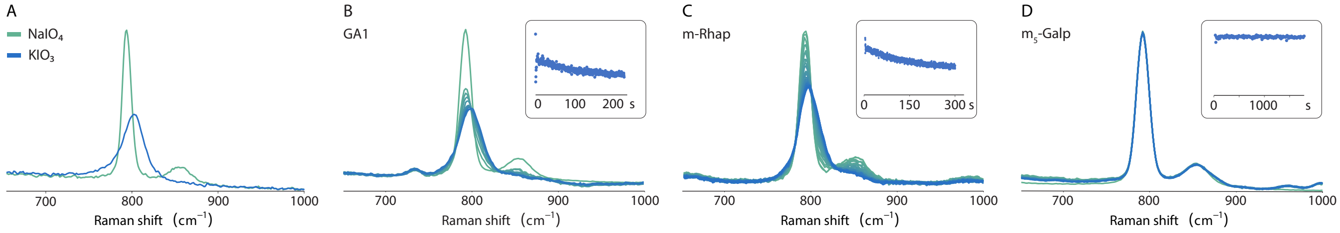

Figure S2: Raman spectra (λ_{exc} 785 nm) of (A) (green) $NaIO_4$ (0.117 M, aq) and (blue) KIO_3 (0.117 M, aq) and (B) GA1 (10 w/v%, aq) with $NaIO_4$ (0.117 M, aq), (C) m-Rhap (0.117 M, aq) with $NaIO_4$ (0.117 M, aq) and (D) m_5-Galp (0.117 M, aq) with $NaIO_4$ (0.117 M, aq) (baseline corrected and corrected for 1:1 dilution). Raman spectra were recorded over time following addition of the respective sugar to a solution of periodate.

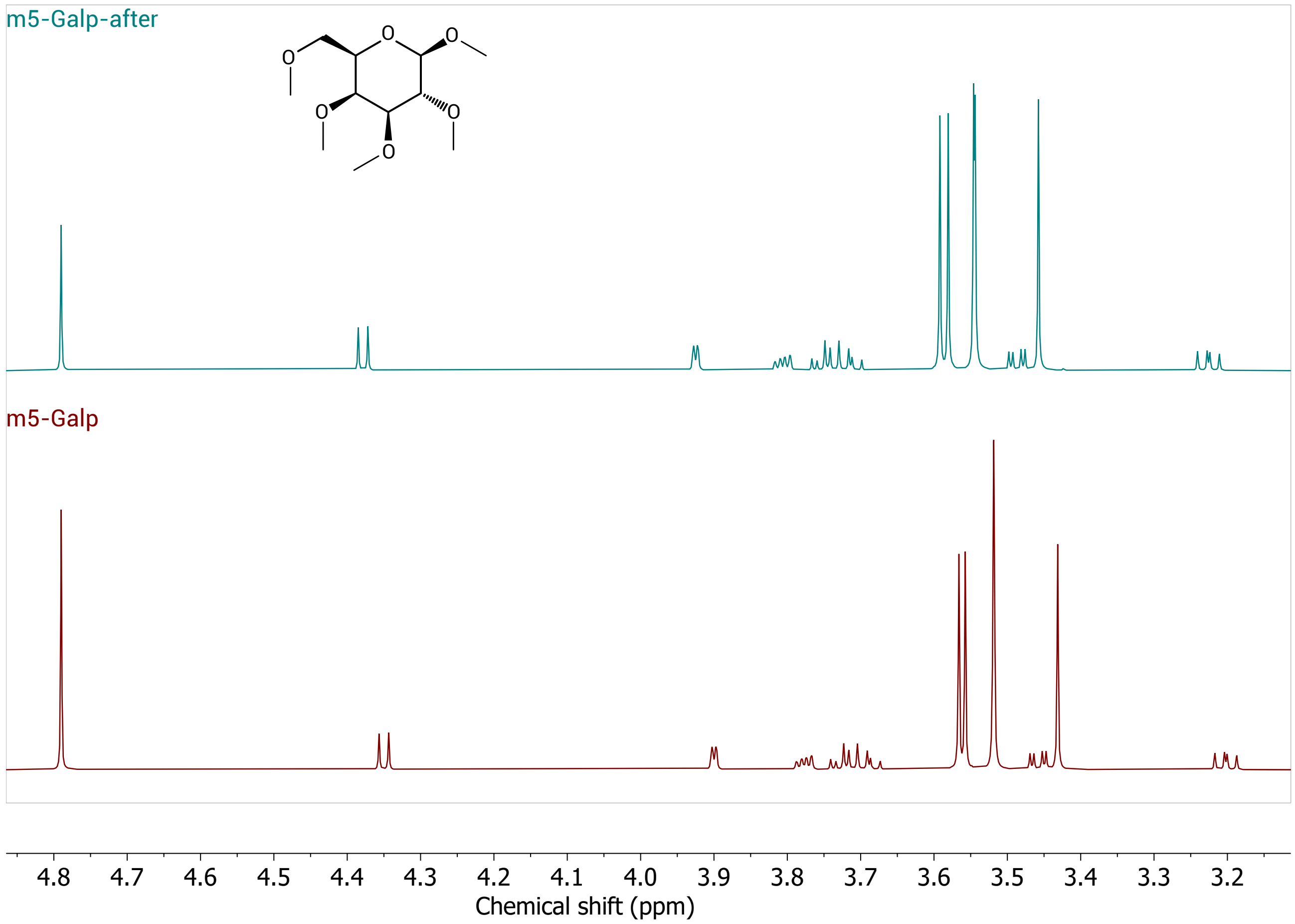

Figure S3: ^{1}H-NMR spectra (600 MHz, D_2O) of m_5-Galp before (bottom) and after (top) mixing with $NaIO_4$ in H_2O, freezedrying and redissolving in D_2O, showing no significant change.

Model peptide

The backbone of the gum arabic polymer consist of a polypeptide chain, the reactivity of which also has to be considered in the oxidation with periodate, especially since reactions have been reported between periodate and amino acids such as serine, threonine, tryptophan, methionine and cysteine.[11] Addition of albumin, a common model protein, to a solution of periodate has no effect on the Raman spectrum of periodate over the time interval typical for sugar oxidation (Fig. S4), indicating a low reactivity of periodate towards polypeptides under these conditions and allows us to assume the minor amount of peptide present in our polymer structure is not oxidised to a significant extent under the present conditions.

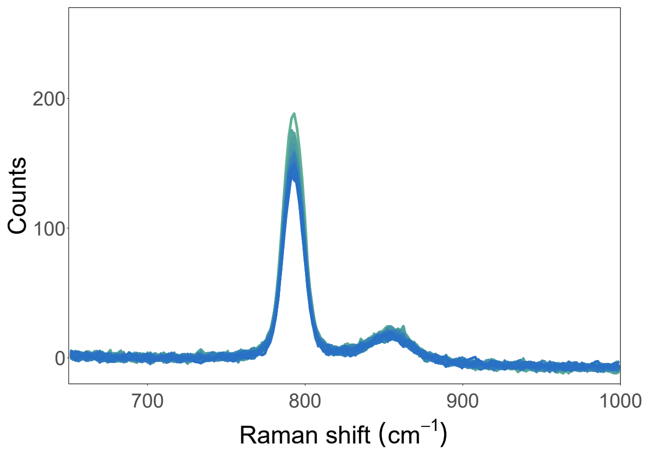

Figure S4: Raman spectra (λ_{exc} 785 nm) of $NaIO_4$ (0.234 M, aq) before (green) and after (green to blue) the addition of albumin (1:1, 2 w/v%, aq), A minor decrease in the periodate band at 792 cm^{-1} is observed, but the band at 854 cm^{-1} is unaffected over 4 min. The initial spectrum of $NaIO_4$ was corrected for dilution and all spectra were baseline corrected.

3. Batch variation in GA1

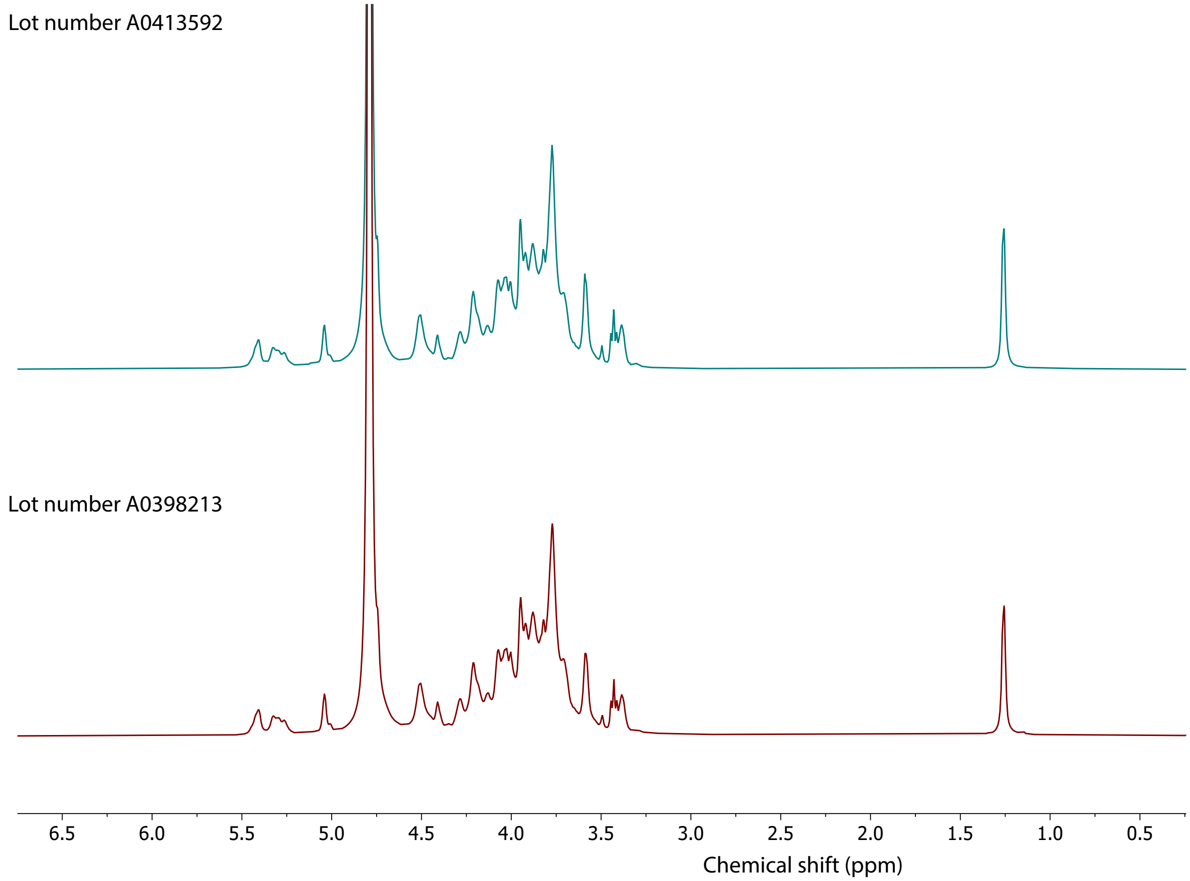

Figure S5: ^{1}H-NMR spectra (600 MHz, D_2O) of two different batches with Lot numbers A0413592 and A0398213 of GA1, showing nearly identical signals.

4. 2D NMR spectra of GA1

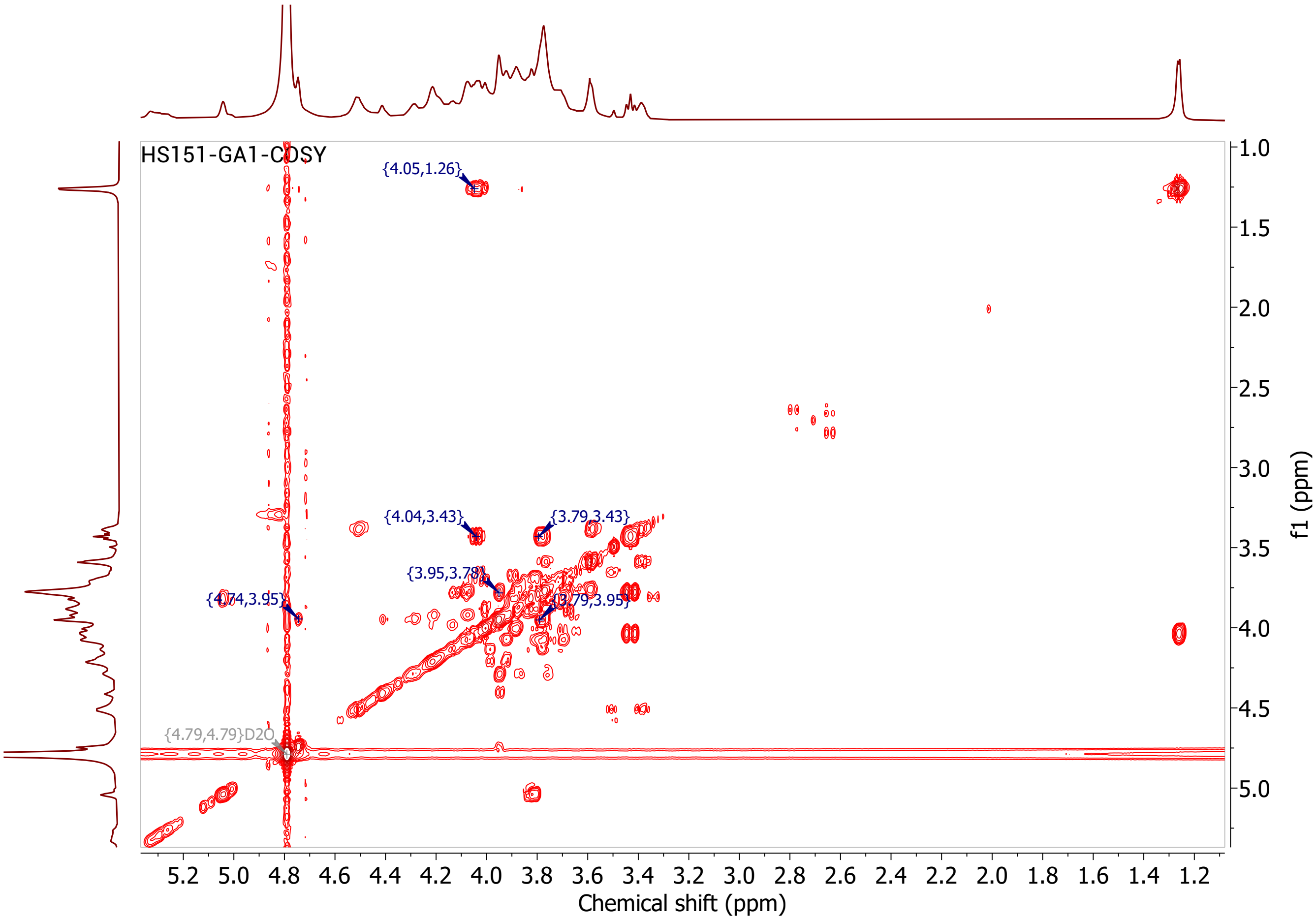

Figure S6: COSY spectrum (600 MHz, D_2O) of GA1, with the signals of 1-Rhap marked. Crosspeaks are present between H1 and H2 (4.74 and 3.95 ppm), H2 and H3 (3.95 and 3.79 ppm), H3 and H4 (3.79 and 3.43 ppm), H4 and H5 (3.43 and 4.04 ppm) and H5 and H6 (4.05 and 1.26 ppm).

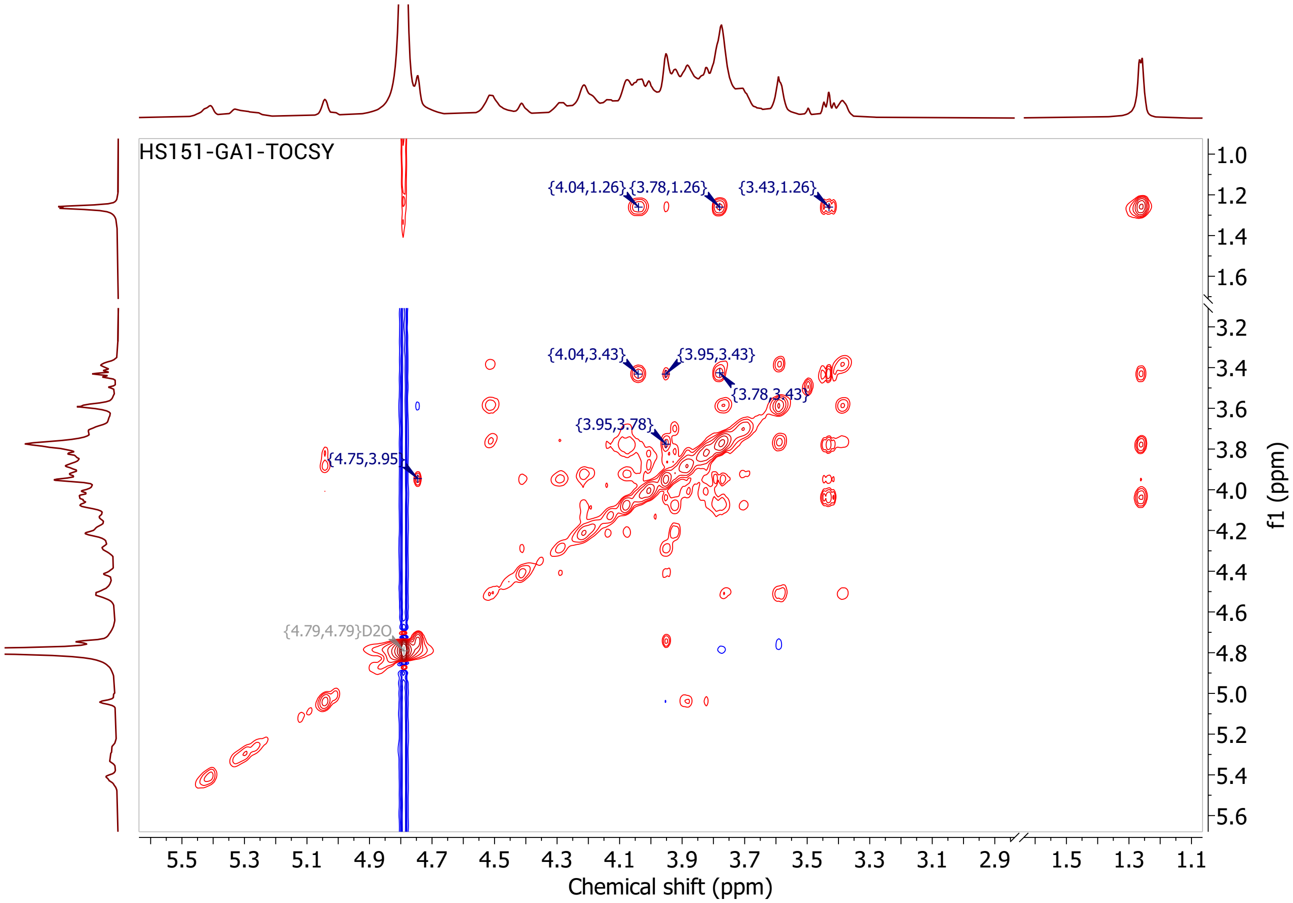

Figure S7: TOCSY spectrum (600 MHz, D_2O) of GA1, with the signals of 1-Rhap marked. Crosspeaks are present between H1 and H2 (4.74 and 3.95 ppm), H2 and H3 (3.95 and 3.79 ppm), H2 and H4 (3.95 and 3.43 ppm), H3 and H4 (3.79 and 3.43 ppm), H3 and H6 (3.79 and 1.26 ppm), H4 and H5 (3.43 and 4.04 ppm), H4 and H6 (3.43 and 1.26 ppm) and H5 and H6 (4.05 and 1.26 ppm).

5. Reproducibility of the oxidation of GA1

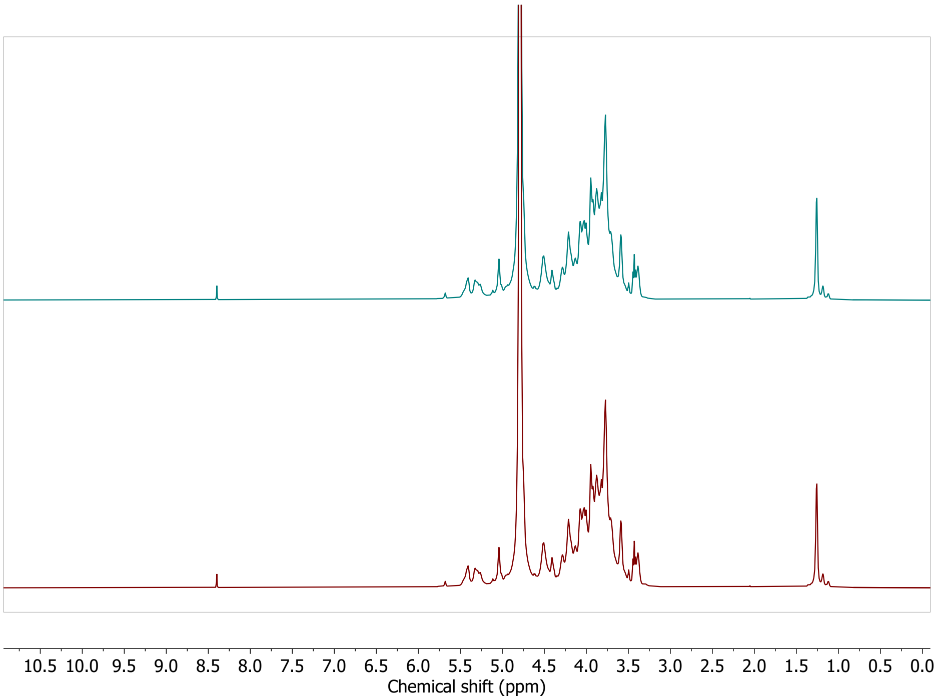

Figure S8: ^{1}H-NMR spectra (600 MHz, D_2O) of GA1 oxidised with 5 % $NaIO_4$. Reactions performed in duplo show reproducibility between spectra.

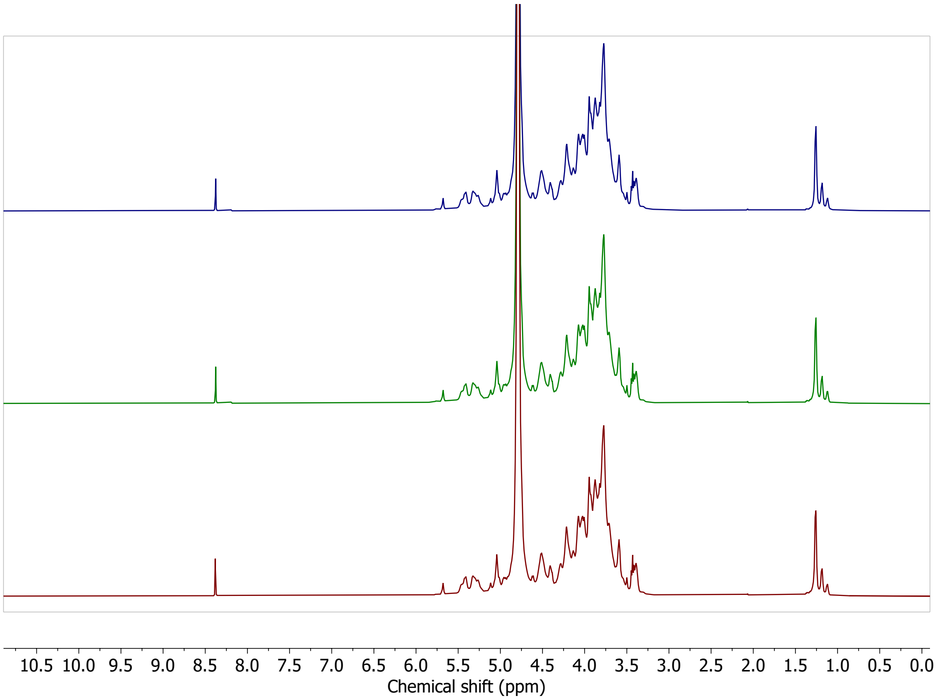

Figure S9: ^{1}H-NMR spectra (600 MHz, D_2O) of GA1 oxidised with 10 % $NaIO_4$. Reactions performed in triplo show reproducibility between spectra.

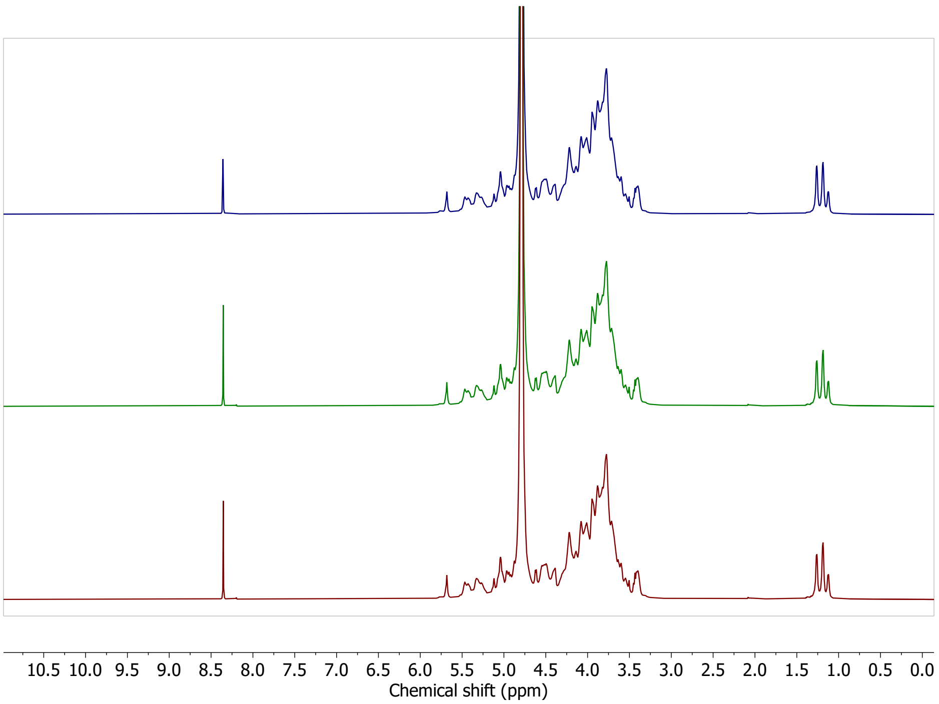

Figure S10: ^{1}H-NMR spectra (600 MHz, D_2O) of GA1 oxidised with 20 % $NaIO_4$. Reactions performed in triplo show reproducibility between spectra.

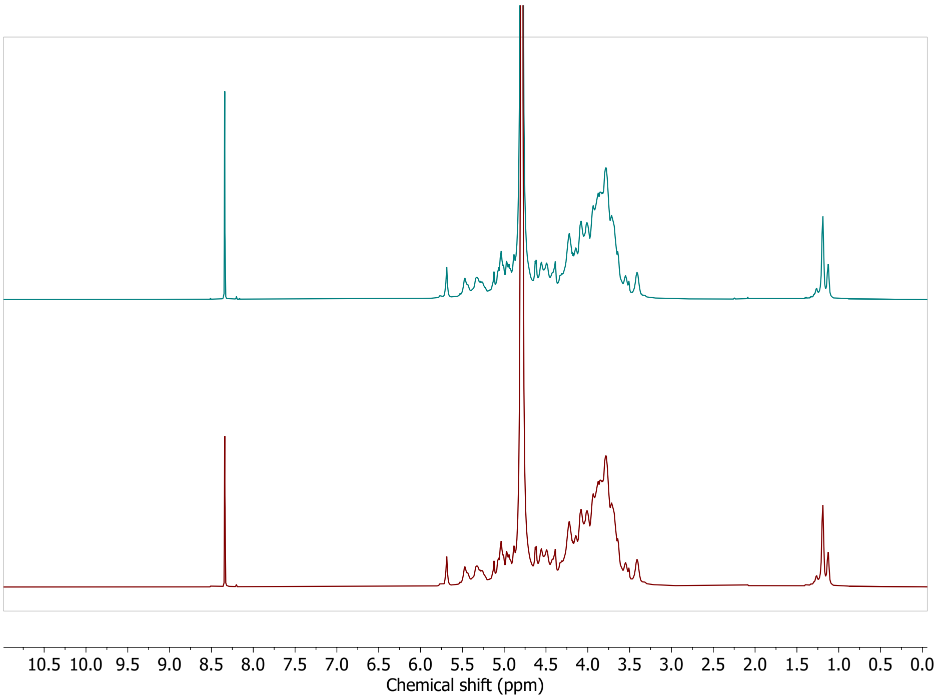

Figure S11: ^{1}H-NMR spectra (600 MHz, D_2O) of GA1 oxidised with 30 % $NaIO_4$. Reactions performed in duplo show reproducibility between spectra.

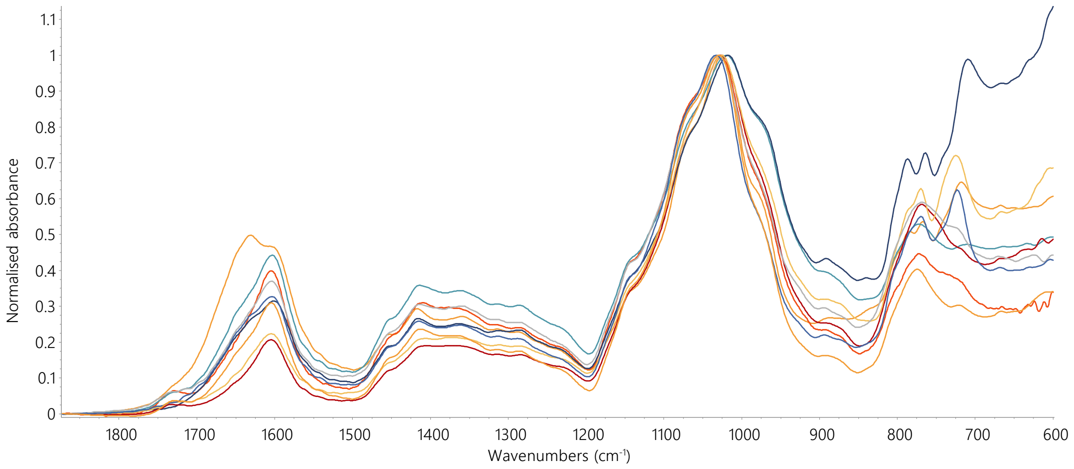

Figure S12: ATR FTIR spectra of freeze-dried GA1 oxidised with 20 % $NaIO_4$, showing overall similar band shapes with differences depending on extent of protonation (in the range 1880-1500 cm^{-1}) and crystal phase of iodate (820-650 cm^{-1}).

6. The oxidation of m-GalpA

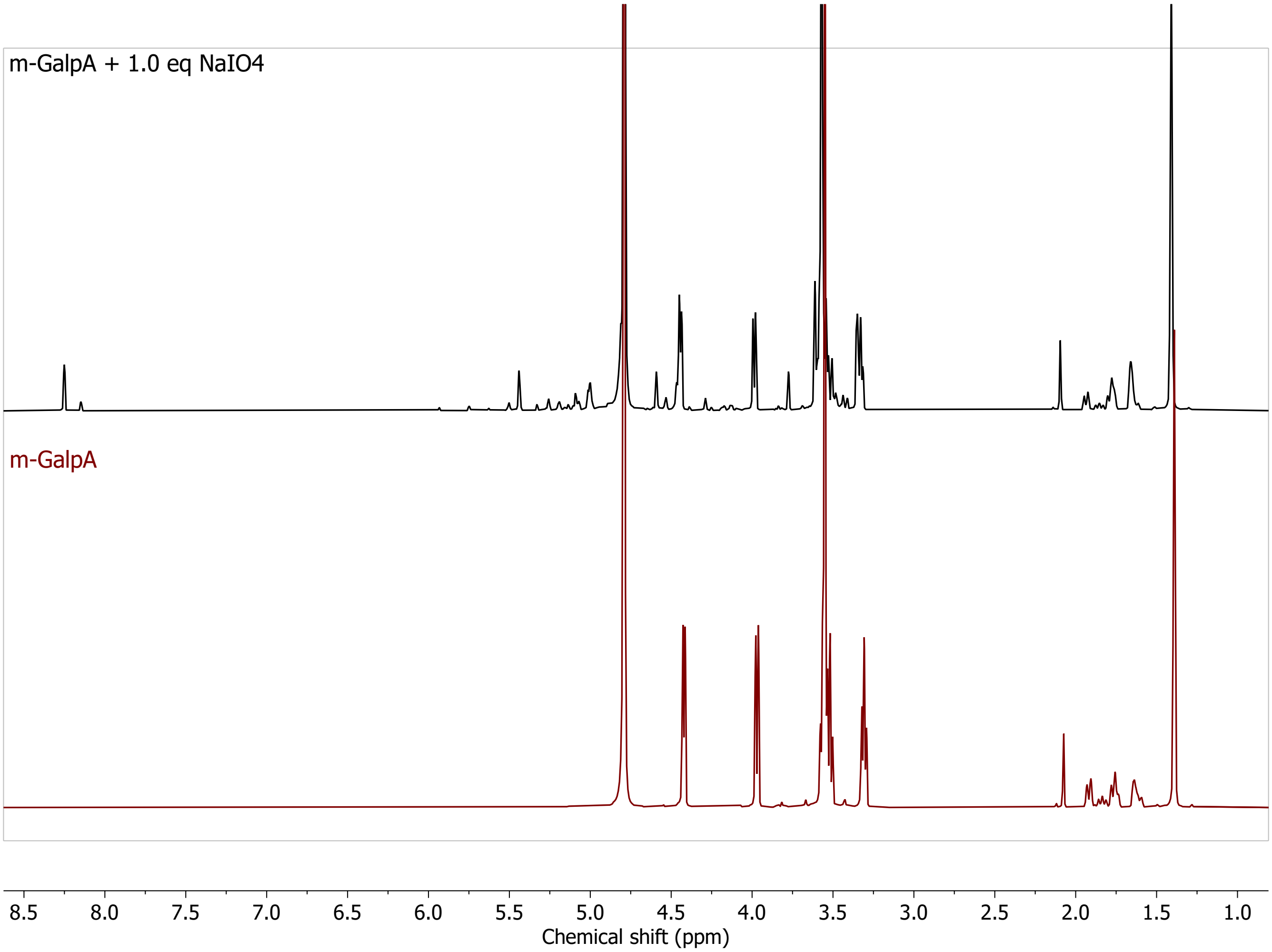

Figure S13: ^{1}H-NMR spectrum (600 MHz, D_2O) of CH_3-β-D-galacturonic acid (m-GalpA) before (bottom) and after (top) oxidation with final concentrations of 0.14 M m-GalpA and 0.14 M periodate. Signals in the range 2.2-1.5 ppm are due to remaining TEMPO used in the synthesis of m-GalpA.

7. Competition experiment m-Rhap and m-GalpA

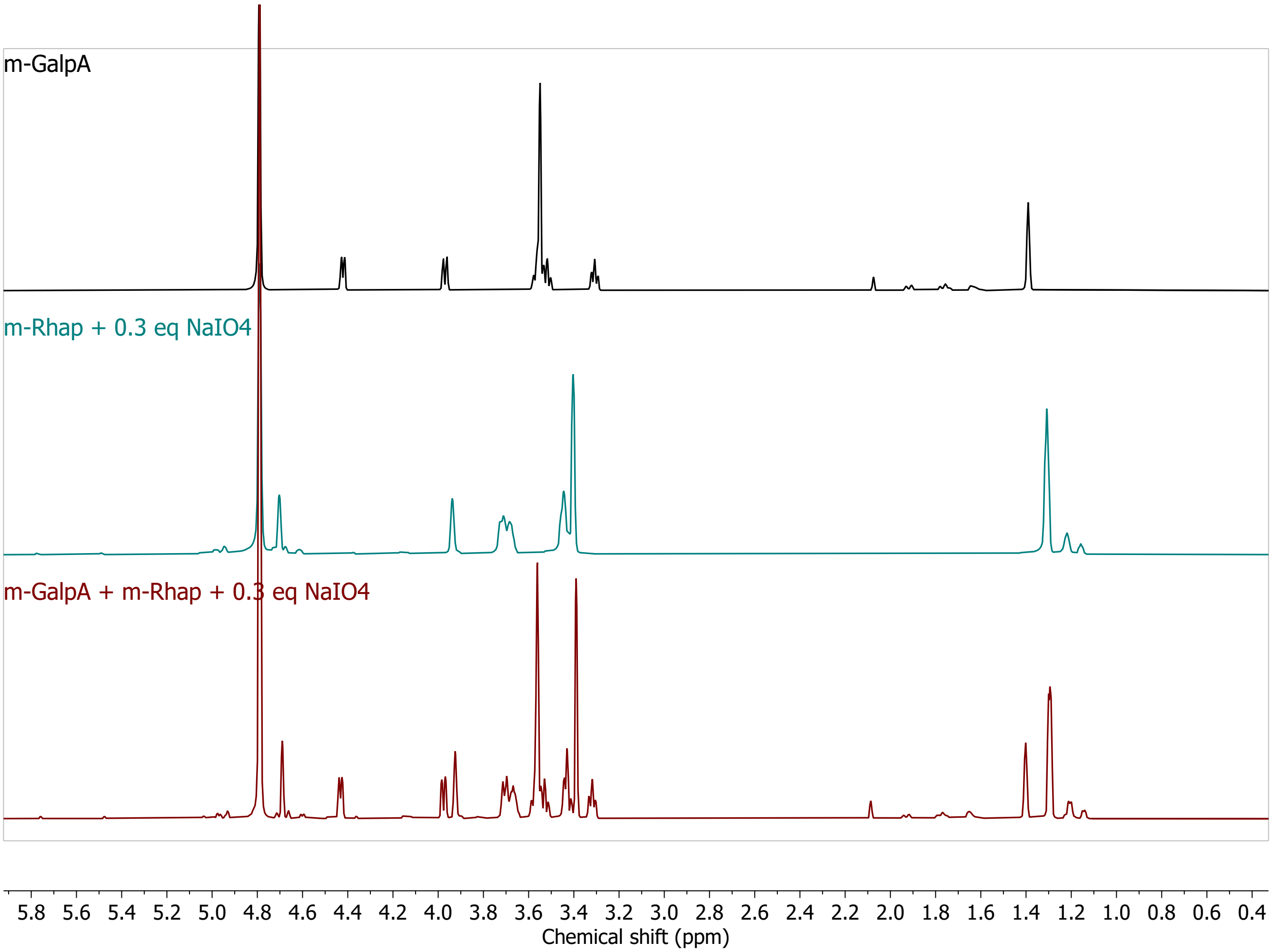

Figure S14: ^{1}H-NMR spectrum (600 MHz, D_2O) of (top) CH_3-β-D-galacturonic acid (m-GalpA) (middle) CH_3-α-L-rhamnopyranoside (m-Rhap) oxidised using 0.3 eq of $NaIO_4$ and (bottom) the competition experiment of m-Rhap and m-Galp with 0.3 eq of $NaIO_4$ showing that the product mixture is unmodified m-GalpA and oxidised m-Rhap.

8. Oxidation of m-Rhap

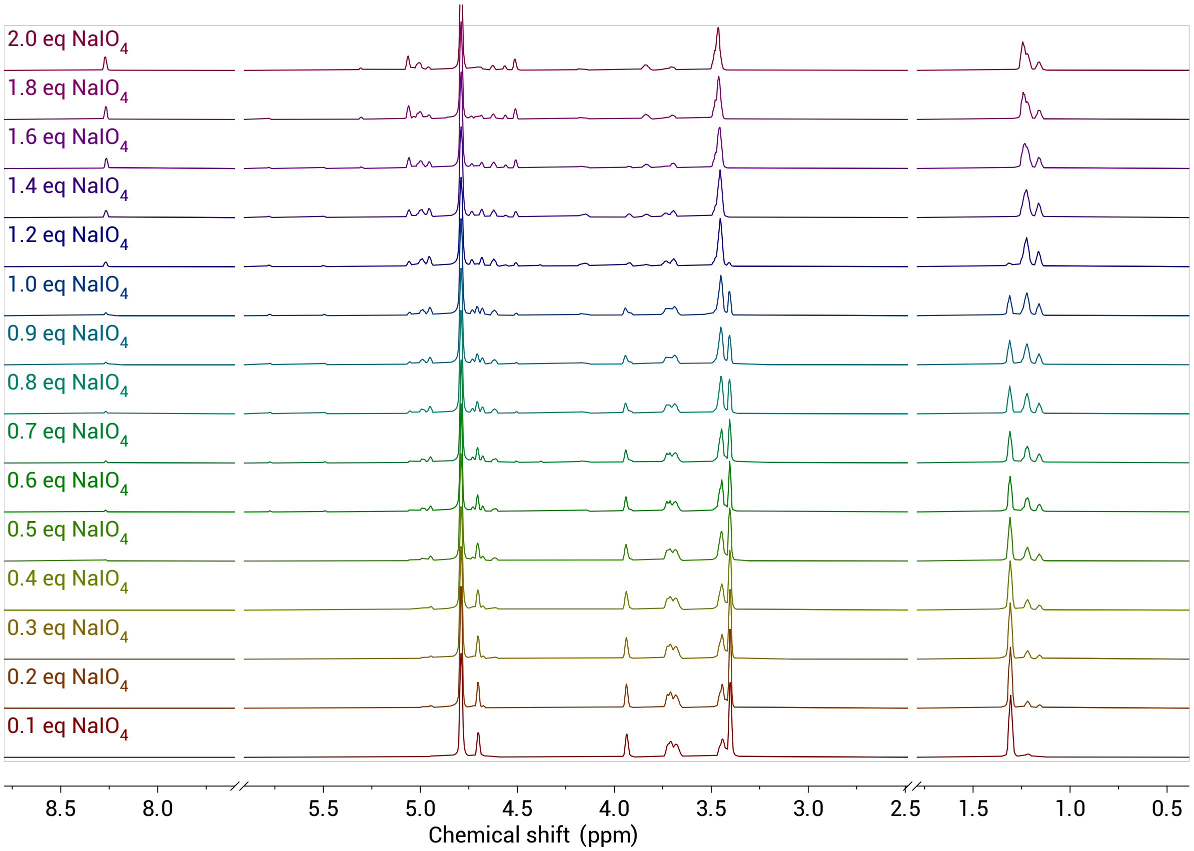

Figure S15: ^{1}H-NMR spectra (600 MHz, D_2O) of m-Rhap (0.28 M, 5 w/v% in D_2O) with various eq. (0.1-2.0, bottom to top, see Table S1) of $NaIO_4$ (0.56 M in D_2O), keeping the total volume constant.

$NaIO_4$ (eq.)	0.28 M m-Rhap (µL)	0.56 M $NaIO_4$ (µL)	D_2O (µL)
0.1	200	10	190
0.2	200	20	180
0.3	200	30	170
0.4	200	40	160
0.5	200	50	150
0.6	200	60	140
0.7	200	70	130
0.8	200	80	120
0.9	200	90	110
1.0	200	100	100
1.2	200	120	80
1.4	200	140	60
1.6	200	160	40
1.8	200	180	20
2.0	200	200	0

Table S1: Amounts used for the data obtained in Figure S15.

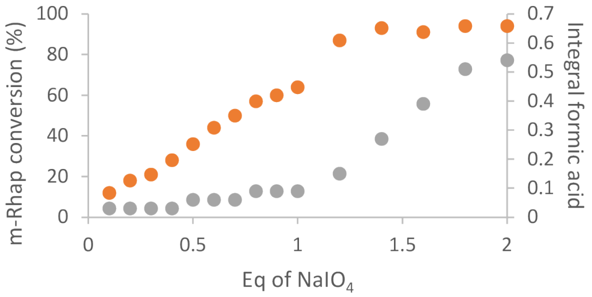

Figure S16: Conversion of m-Rhap (orange) determined from the integral of its signals due to methyl protons and the integral of the formic acid signal (grey) vs equivalents of $NaIO_4$ used.

9. Traces of aldehyde

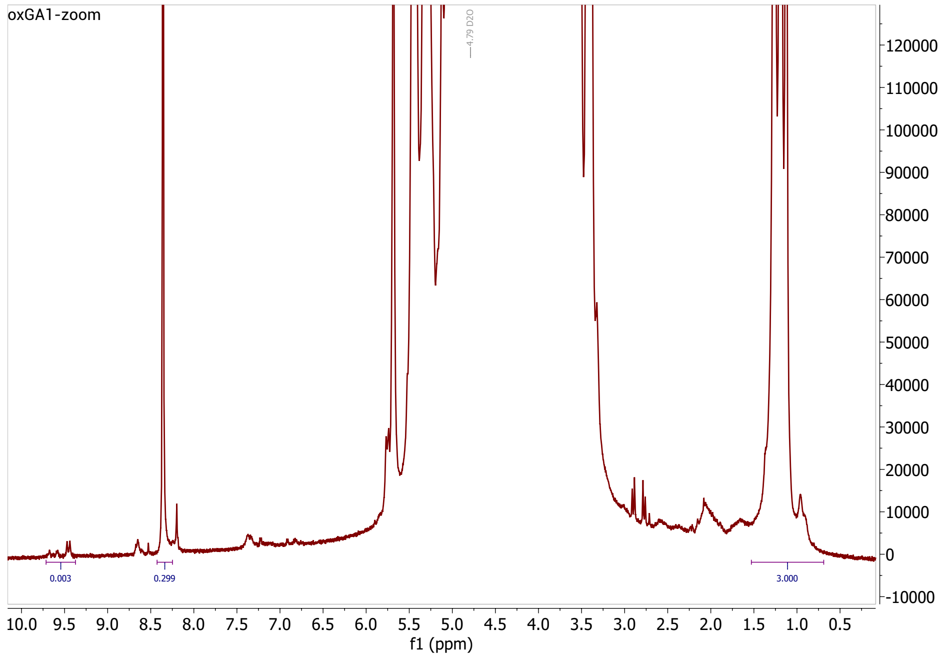

Figure S17: ^{1}H-NMR spectrum of oxGA1 (600 MHz, 10 w/v% in D_2O with 0.2 eq of $NaIO_4$) showing minor aldehyde signals, which integrate as 0.003 relative to the three methyl protons of Rhap units present. The signal at 8.36 ppm is formic acid.

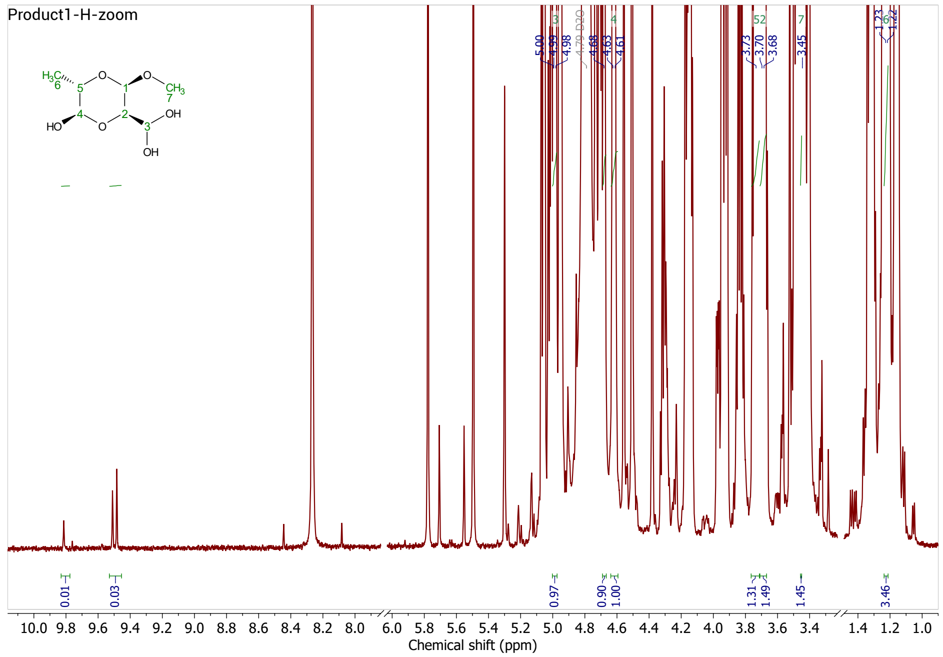

Figure S18: ^{1}H-NMR spectrum of oxidised m-Rhap (600 MHz, 0.234 M in D_2O with 1.0 eq of $NaIO_4$) showing minor aldehyde signals, which integrate as 0.04 per product **1** molecule. The signal at 8.26 ppm is formic acid.

10. Oxidation of m-Rhap

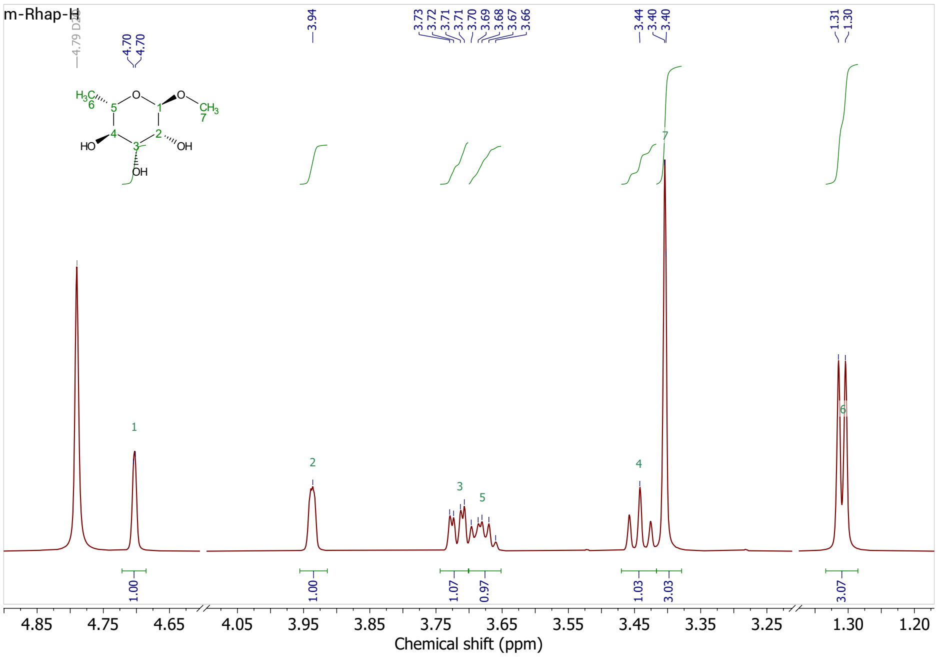

Figure S19: ^{1}H-NMR spectrum of CH_3-α-L-rhamnopyranoside (m-Rhap) (600 MHz, D_2O): 1.31 (d, J = 6.33 Hz, 3H, H6); 3.40 (s, 3H, H7); 3.44 (t, J = 9.6 Hz, 1H, H4); 3.68 (m, J = 9.7, 6.33 Hz, 1H, H5); 3.72 (dd, J = 9.7, 3.0 Hz, 1H, H3); 3.94 (dd, J = 1.4, 3.0 Hz, 1H, H2); 4.70 (s, 1H, H1). Data matches reported values.[12]

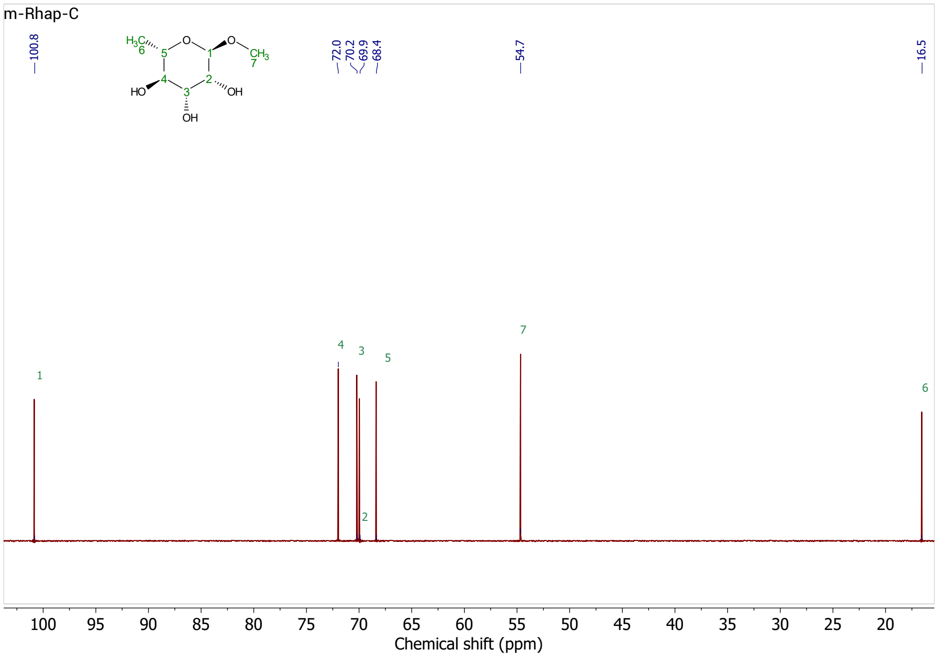

Figure S20: ^{13}C-NMR spectrum of CH_3-α-L-rhamnopyranoside (m-Rhap) (125 MHz, D_2O): 16.5 (C6); 54.7 (C7); 68.4 (C5); 69.9 (C2); 70.2 (C3); 18.0 (C4); 100.8 (C1).

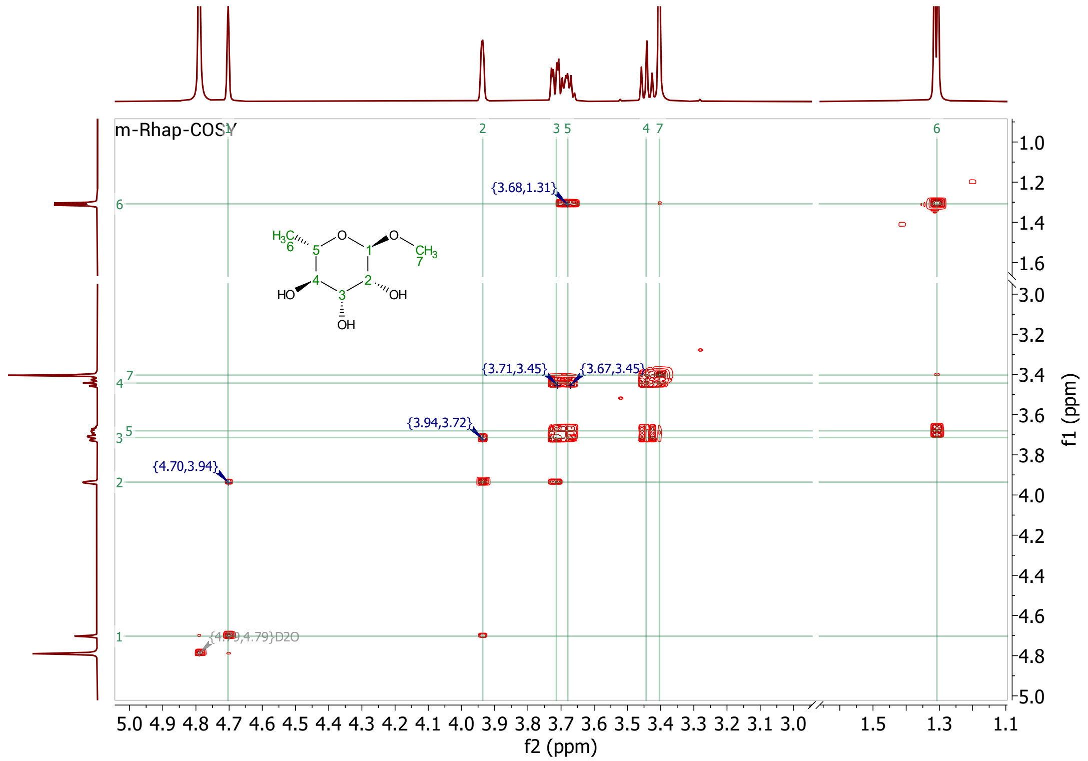

Figure S21: COSY NMR spectrum of CH_3-α-L-rhamnopyranoside (m-Rhap), showing crosspeaks between H1 and H2 (4.70 and 3.94 ppm), H2 and H3 (3.94 and 3.72 ppm), H3 and H4 (3.71 and 3.45 ppm), H4 and H5 (3.45 and 3.67 ppm), and H5 and H6 (3.68 and 1.31 ppm).

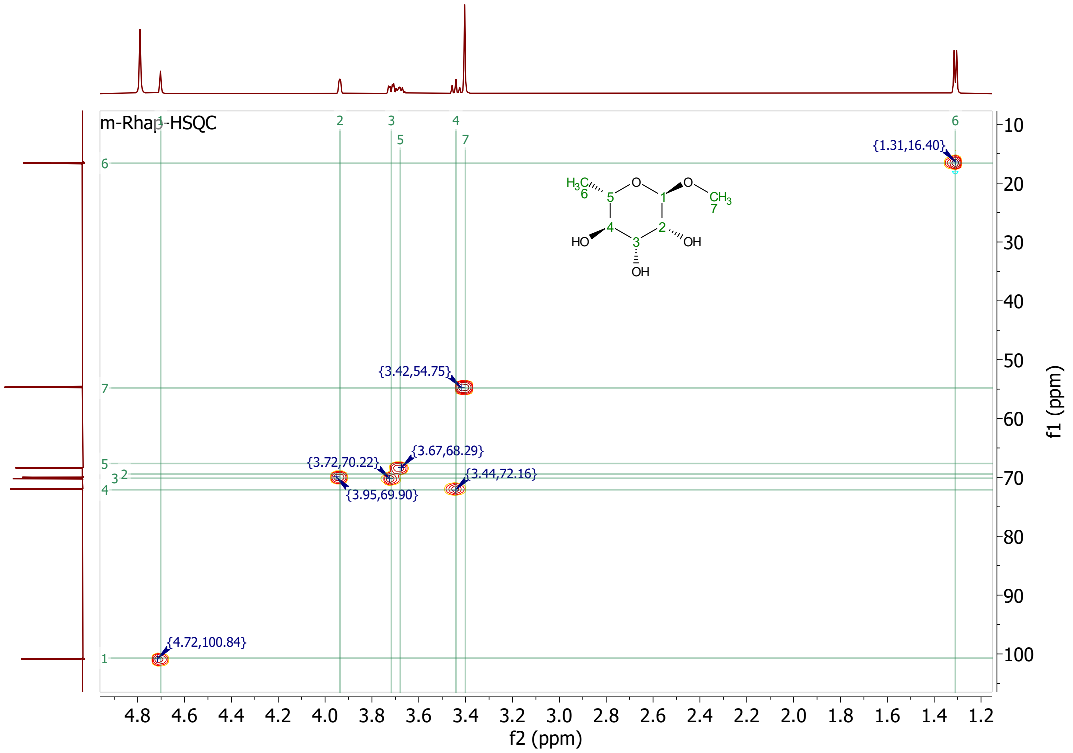

Figure S22: HSQC NMR spectrum of CH_3-α-L-rhamnopyranoside (m-Rhap), showing crosspeaks between H1 and C1 (4.72 and 100.8 ppm), H2 and C2 (3.95 and 69.9 ppm), H3 and C3 (3.72 and 70.2 ppm), H4 and C4 (3.44 and 72.2 ppm), H5 and C5 (3.67 and 68.3 ppm), H6 and C6 (1.31 and 16.4 ppm), and H7 and C7 (3.42 and 54.8 ppm).

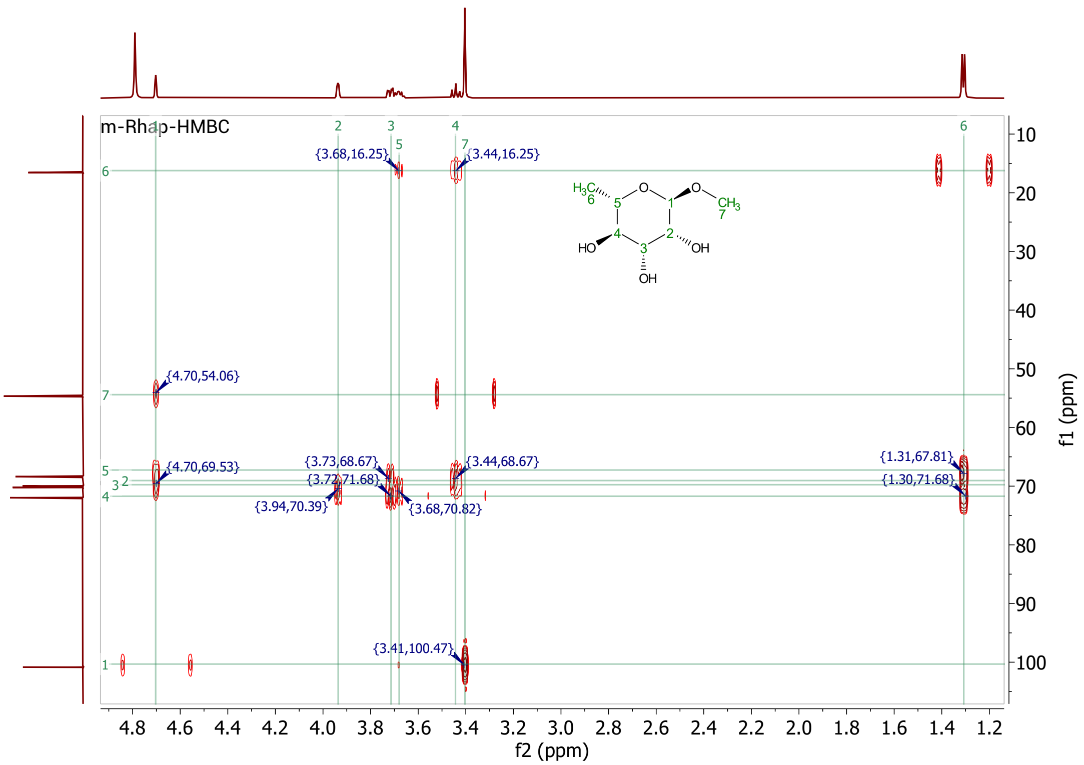

Figure S23: HMBC spectrum (600 MHz, D_2O) of CH_3-α-L-rhamnopyranoside (m-Rhap), showing crosspeaks between H1 and C7 (4.70 and 54.1 ppm), H1 and C2 (4.70 and 69.5 ppm), H2 and C3 (3.94 and 70.4 ppm), H3 and C5 (3.73 and 68.7 ppm), H3 and C4 (3.72 and 71.7 ppm), H4 and C6 (3.44 and 16.3 ppm), H4 and C3 (3.44 and 68.7 ppm), H5 and C3/C4 (3.68 and 70.8 ppm), H5 and C6 (3.68 and 16.3 ppm), H6 and C5 (1.31 and 67.8 ppm), H6 and C4 (1.30 and 71.7 ppm), and H7 and C1 (3.41 and 100.5 ppm).

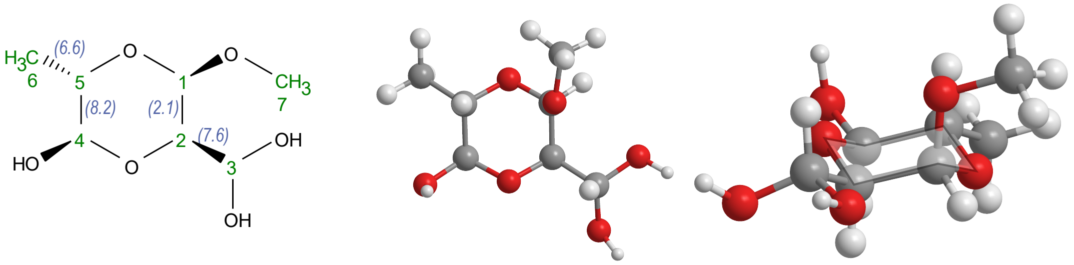

Figure S24: Structure of product **1** with between brackets the J-coupling values of H1, H2, H3, H4, H5 and H6 as obtained from the ^{1}H-NMR spectrum (Fig. S26), which were used to determine the stereochemistry of product **1**. DFT calculations on all four possible products obtained by changing the stereochemistry on the C2 and C4, show that for each of them the lowest energy state of the molecule is when it adopts a chair conformation. The large J-coupling of 8.2 Hz between H4 and H5 of product **1** indicates an axial-axial interaction,[13] and with H5 axial pointing up, this means that H4 has to be axial pointing down and the hydroxyl group on the C4 atom has to be pointing up in an equatorial position. The small J-coupling of 2.1 Hz between H1 and H2 indicates either an equatorial-equatorial or an axial-equatorial coupling.[13] Considering the way in which this product is formed (Fig. S25), the only option for intramolecular ring-closing to happen is for the OH on the C2 to attack the C4. This results in in the C3 substituent on the C2 position pointing up, provided enolisation does not occur. With both the OMe on the C1 pointing up and the substituent on the C3 pointing up, whether they are axial or equatorial depends on whether the molecule adopts a boat or chair conformation. In the (most stable) chair conformation, the OMe on the C1 is axial and the substituent on the C2 is oriented equatorially. As a result, H1 and H2 are equatorial and axial, respectively. Hence, the small coupling constant of 2.1 Hz between H1 and H2 is due to an equatorial-axial interaction.

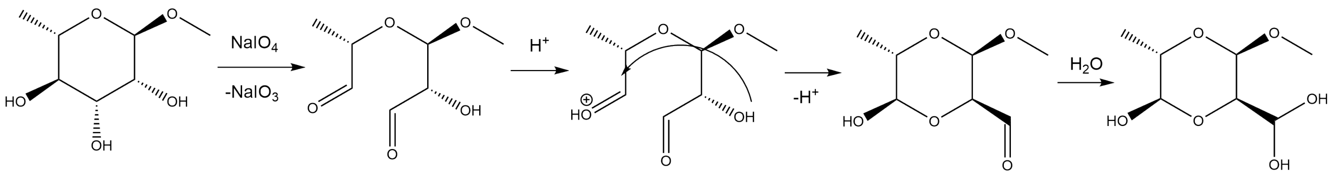

Figure S25: Proposed mechanism for the formation of product **1**. First, oxidation by periodate occurs on the anti diol of m-Rhap, forming an aldehyde on the C3 and C4 positions. Protonation of the aldehyde on the C4 position, followed by the attack of the C2 hydroxyl group and subsequent deprotonation, leads to ring-closing and the formation of a substituted dioxane, still containing one aldehyde on the C3. Hydration leads to the formation of product **1**.

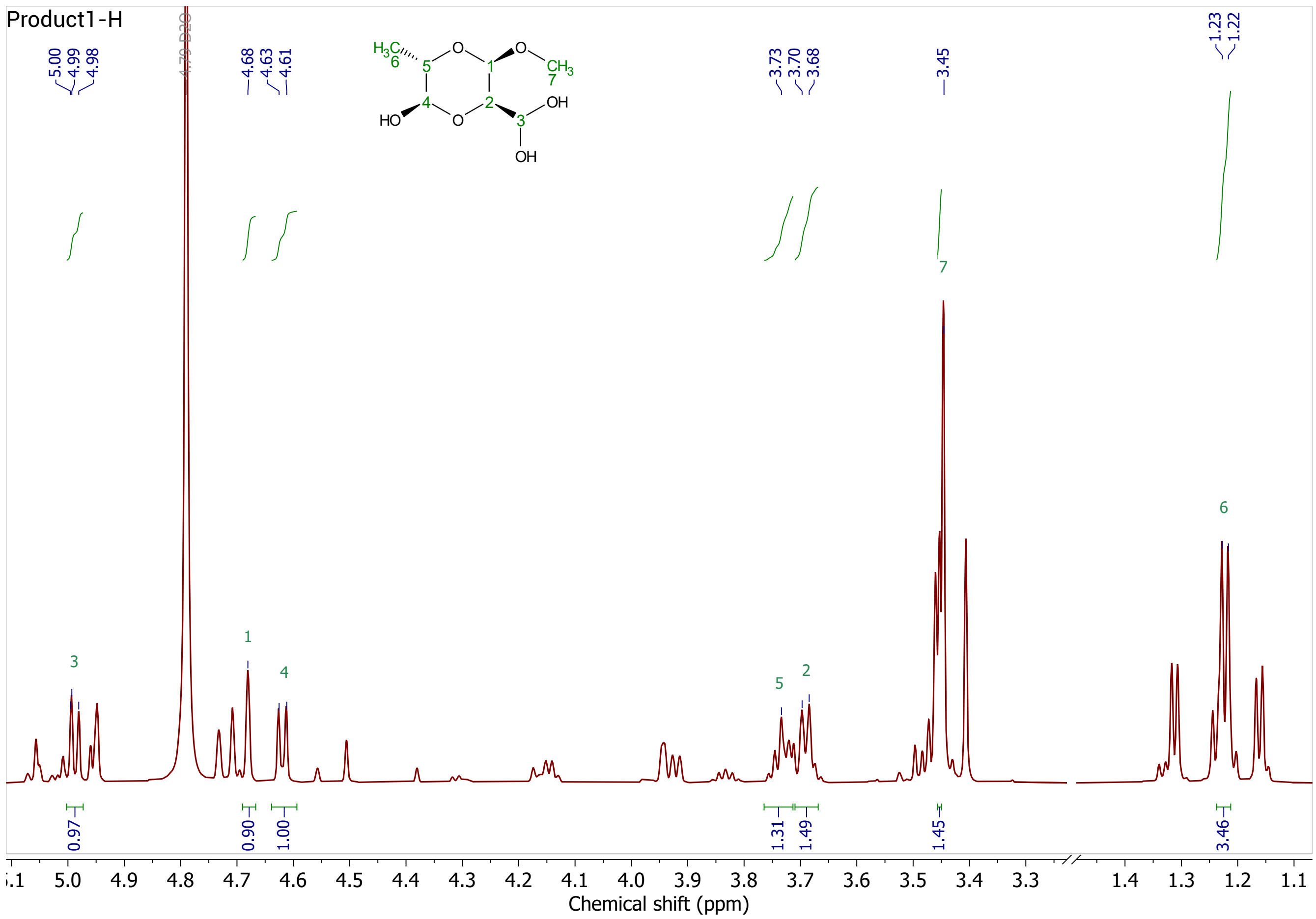

Figure S26: ^{1}H-NMR spectrum (600 MHz, D_2O) of oxidised CH_3-α-L-rhamnopyranoside (m-Rhap) using 1.0 eq of periodate, showing the signals for product **1**: 1.23 (d, J = 6.6 Hz, 3H, H6); 3.45 (s, 3H, H7); 3.69 (dd, J = 2.1, 7.6 Hz, 1H, H2); 3.73 (dq, J = 8.2, 6.6 Hz, 1H, H5); 4.62 (d, J = 8.2 Hz, 1H, H4); 4.68 (d, J = 2.1 Hz, 1H, H1); 4.99 (d, J = 7.6 Hz, 1H, H3).

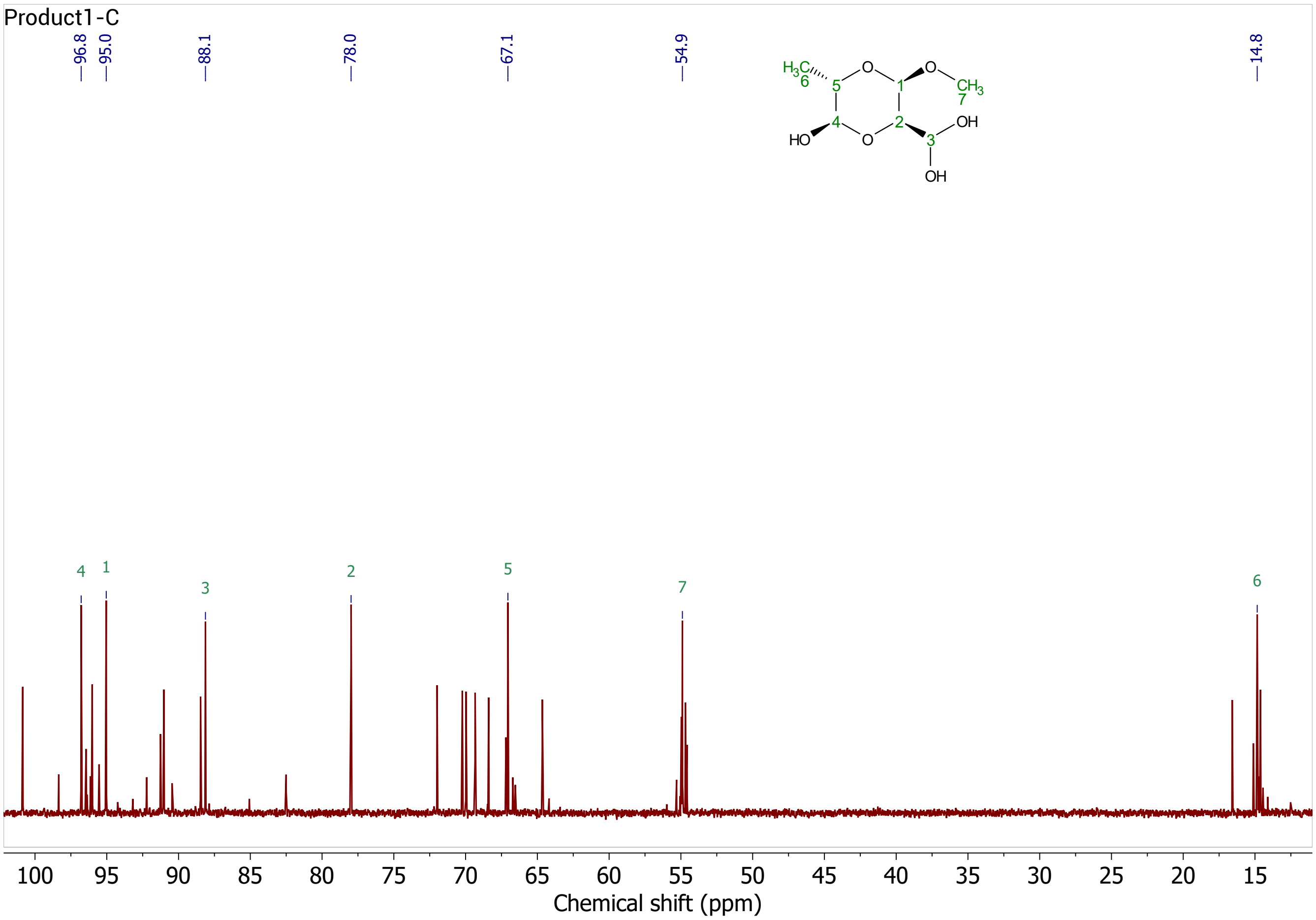

Figure S27: ^{13}C-NMR spectrum (125 MHz, D_2O) of oxidised CH_3-α-L-rhamnopyranoside (m-Rhap) using 1.0 eq of periodate, showing the signals for product **1**: 14.9 (C6); 54.9 (C7); 67.1 (C5); 78.0 (C2); 88.1 (C3); 95.0 (C1); 96.8 (C4) ppm.

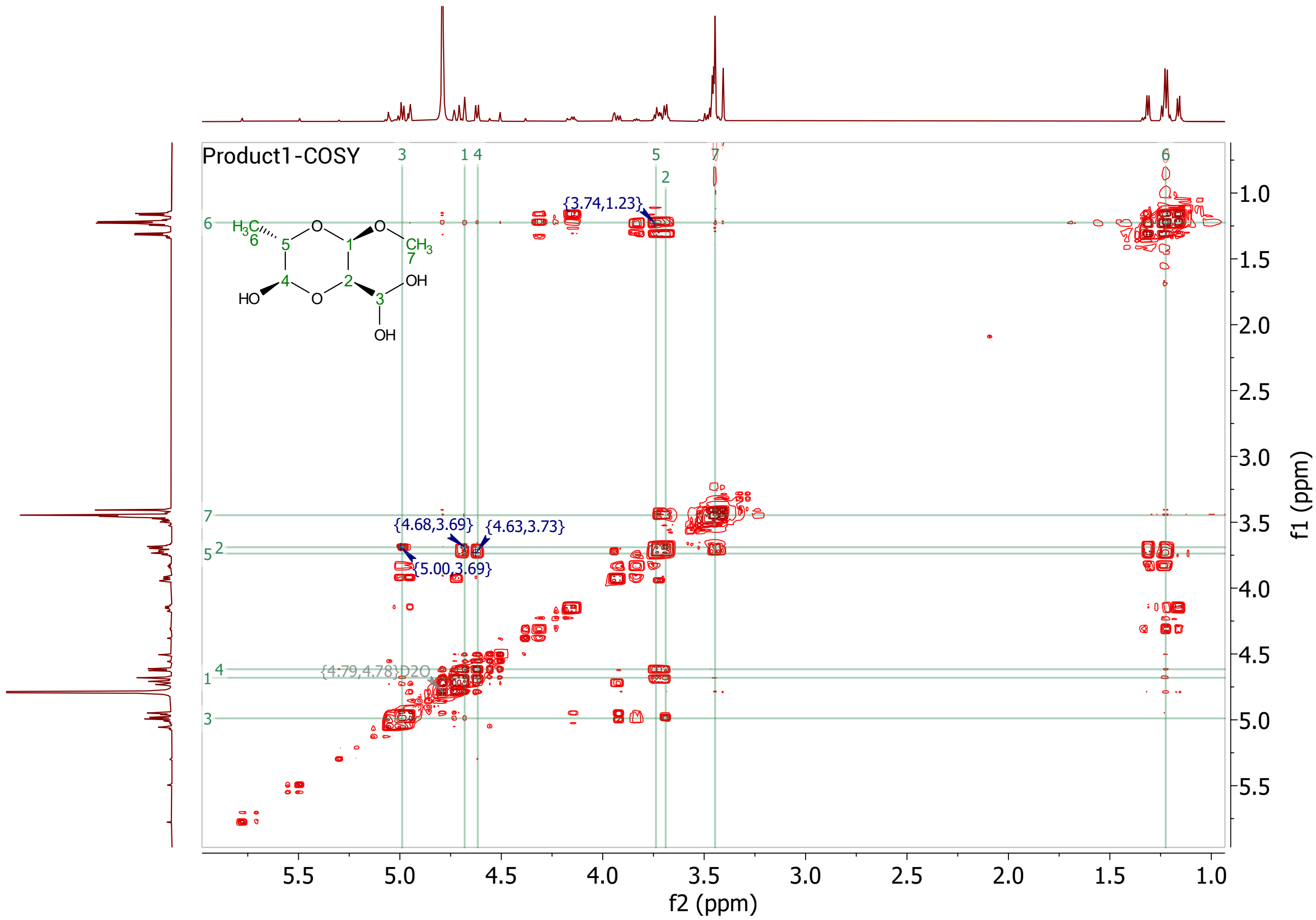

Figure S28: COSY spectrum (600 MHz, D_2O) of oxidised CH_3-α-L-rhamnopyranoside (m-Rhap) using 1.0 eq of periodate, showing the signals for product **1** with crosspeaks between H1 and H2 (4.68 and 3.69 ppm), H2 and H3 (3.69 and 5.00 ppm), H4 and H5 (4.63 and 3.73 ppm), and H5 and H6 (3.74 and 1.23 ppm). Additional weak crosspeaks are present between H1 and H6, H4 and H6, H7 and H6, and H1 and H3 (not marked).

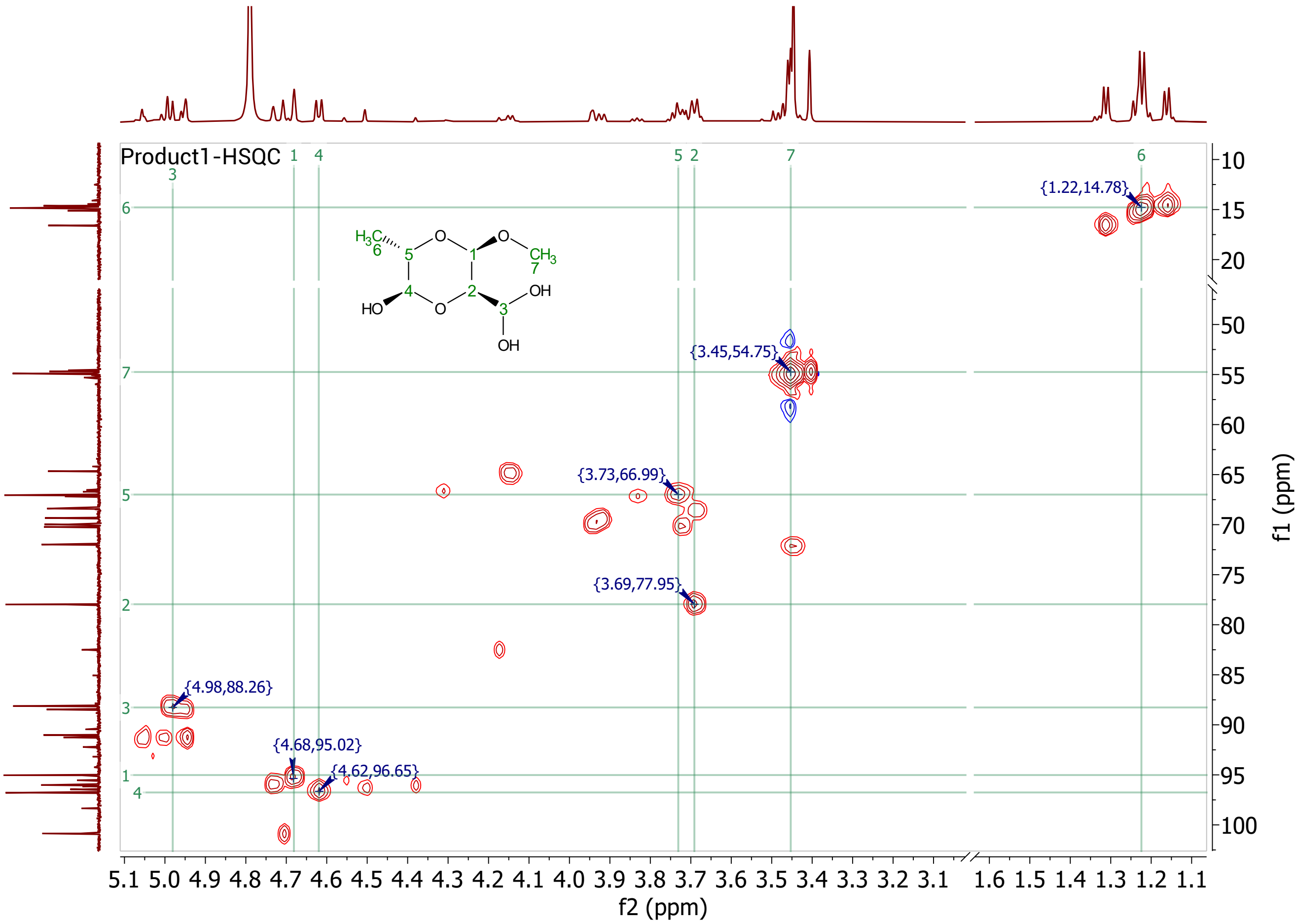

Figure S29: HSQC spectrum (600 MHz, D_2O) of oxidised CH_3-α-L-rhamnopyranoside (m-Rhap) using 1.0 eq of periodate, showing the signals for product **1**, with crosspeaks between H1 and C1 (4.68 and 95.0 ppm), H2 and C2 (3.69 and 78.0 ppm), H3 and C3 (4.98 and 88.3 ppm), H4 and C4 (4.62 and 96.7 ppm), H5 and C5 (3.73 and 67.0 ppm), H6 and C6 (1.22 and 14.8 ppm), and H7 and C7 (3.45 and 54.8 ppm).

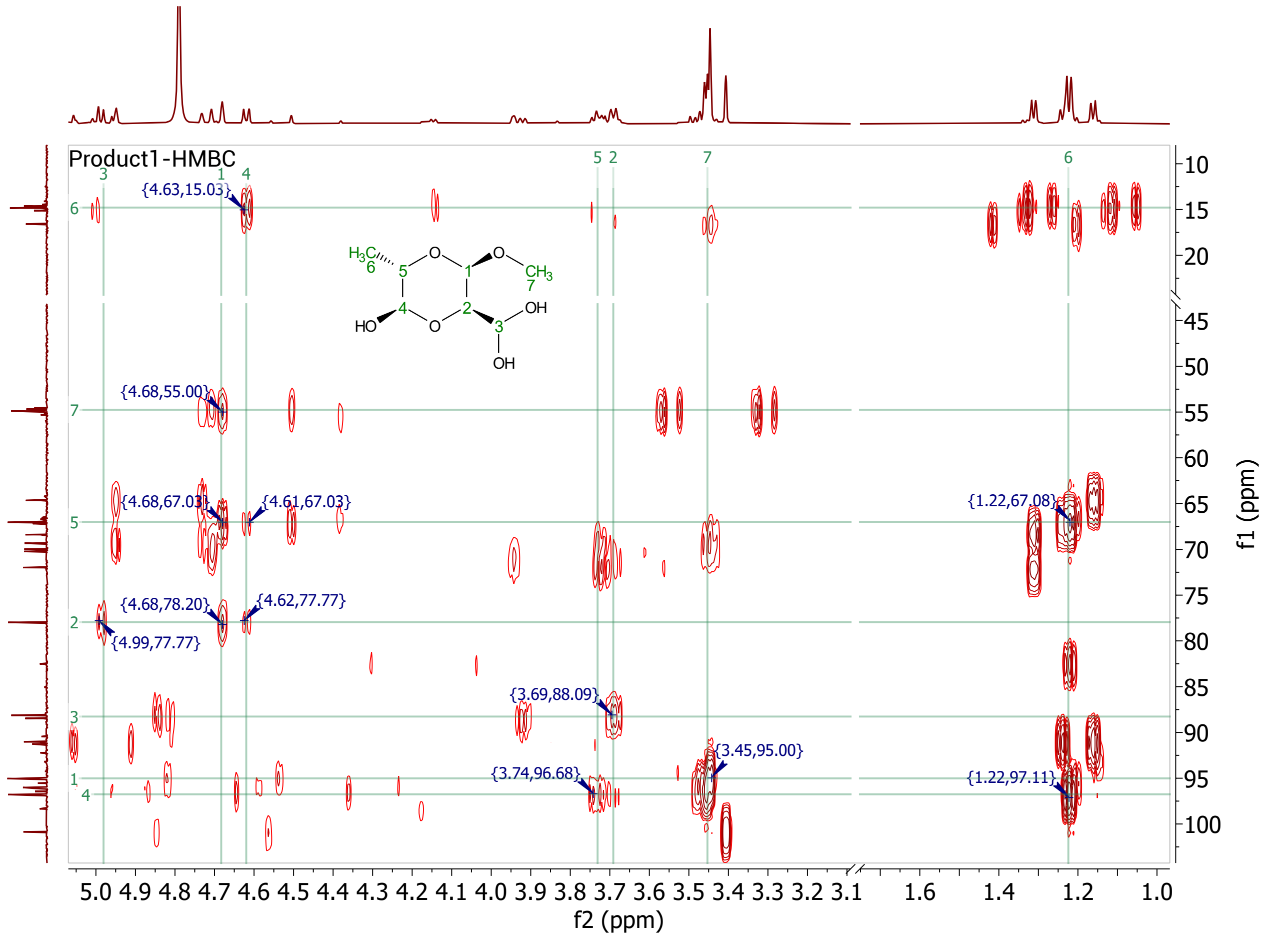

Figure S30: HMBC spectrum (600 MHz, D_2O) of oxidised CH_3-α-L-rhamnopyranoside (m-Rhap) using 1.0 eq of periodate, showing the signals for product **1**, with crosspeaks between H1 and C2 (4.68 and 78.2 ppm), H1 and C5 (4.68 and 67.0 ppm), H1 and C7 (4.68 and 55.0 ppm), H2 and C3 (4.69 and 88.1 ppm), H3 and C2 (4.99 and 77.8 ppm), H4 and C2 (4.62 and 77.8 ppm), H4 and C5 (4.61 and 67.0 ppm), H4 and C6 (4.63 and 15.0 ppm), H5 and C4 (3.74 and 96.7 ppm), H6 and C4 (1.22 and 97.1 ppm), and H6 and C5 (1.22 and 67.1 ppm).

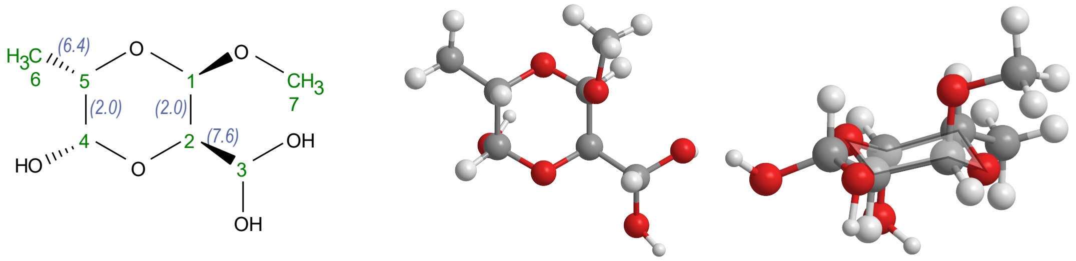

Figure S31: (left) Structure of product **2** with between brackets the J-coupling values of H1, H2, H3, H4, H5 and H6 as obtained from the ^{1}H-NMR spectrum (Fig. S33), which were used to determine the stereochemistry of product **2**. Similar to the reasoning for product 1 (Fig. S24), the small J-coupling of 2.1 Hz between H1 and H2 indicates axial-equatorial coupling.[13] With H1 being equatorial, this means that H2 has to be oriented axial, and pointing down, and the substituent on the C2 has to be pointing up. The additional small J-coupling of 2.0 Hz between H4 and H5 indicates another axial-equatorial interaction,[13] and with H5 in the axial position, this means that H4 has to be equatorial and pointing up, with the hydroxyl group on the C4 atom pointing down. The different J-coupling between H4 and H5 shows the different stereochemistry on the C4 position in products **1** and **2**. (middle, right) Calculated lowest energy structure for product **2**, showing the axial and equatorial positions and the chair conformation.

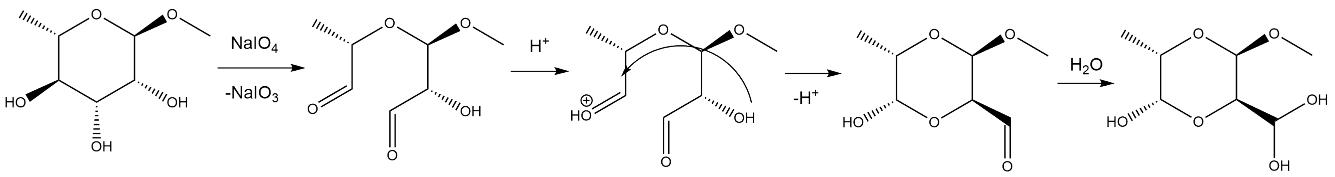

Figure S32: Proposed mechanism for the formation of product **2**. First, oxidation by periodate occurs on the anti diol of m-Rhap, forming an aldehyde on the C3 and C4 positions. Protonation of the aldehyde on the C4 position, followed by the attack of the C2 hydroxyl group and subsequent deprotonation, leads to ring-closing and the formation of a substituted dioxane, still containing one aldehyde on the C3. Hydration leads to the formation of product **2**.

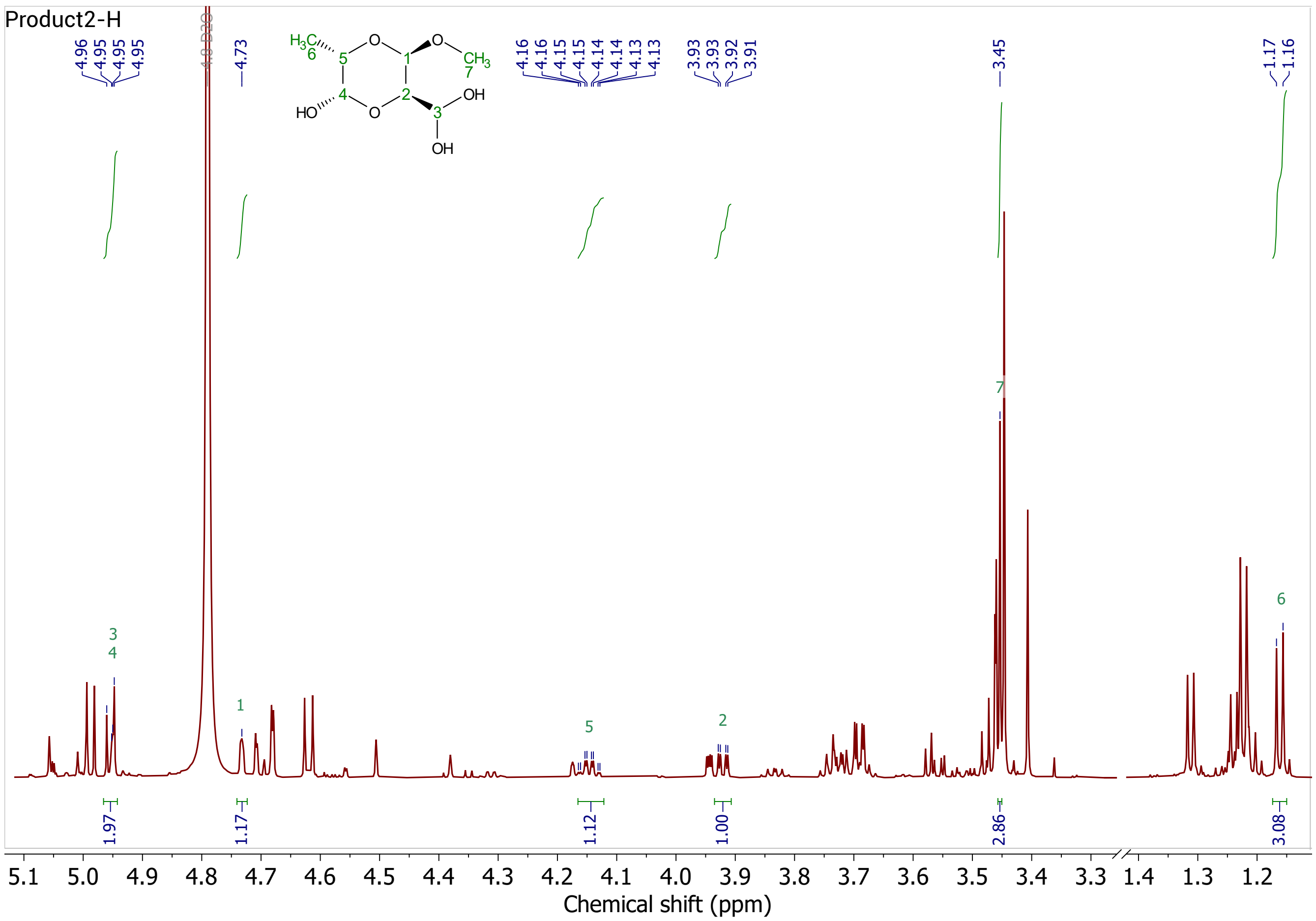

Figure S33: ^{1}H-NMR spectrum (600 MHz, D_2O) of oxidised CH_3-α-L-rhamnopyranoside (m-Rhap) using 1.0 eq of periodate, showing the signals for product **2**: 1.17 (d, J = 6.4 Hz, 3H, H6); 3.45 (s, 3H, H7); 3.93 (dd, J = 2.0, 7.6 Hz, 1H, H2); 4.15 (dq, J = 2.0, 6.4 Hz, 1H, H5); 4.73 (d, J = 2.0 Hz, 1H, H1); 4.95 (d, J = 7.6 Hz, 1H, H3); 4.95 (d, J = 2.0 Hz, 1H, H4).

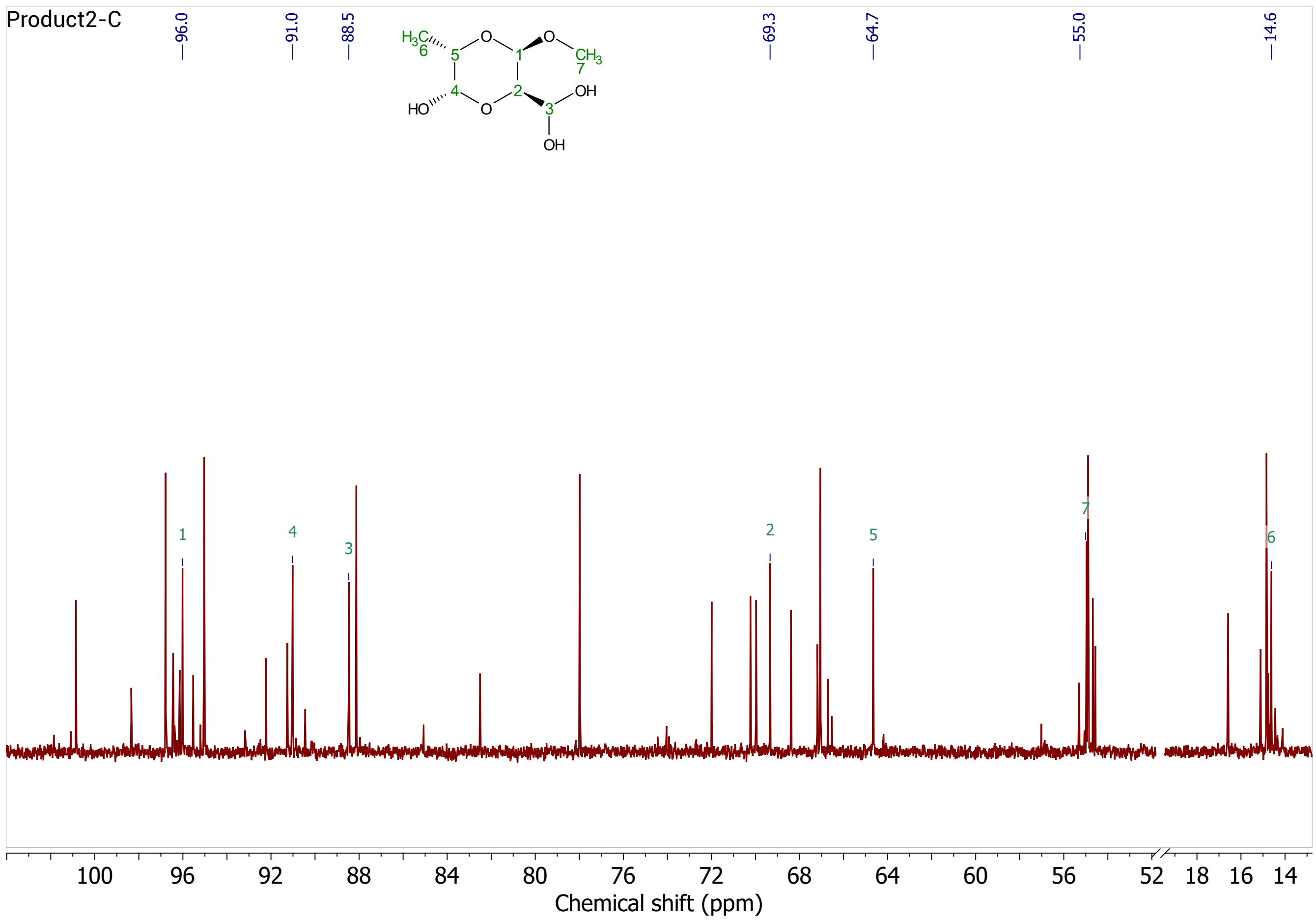

Figure S34: ^{13}C-NMR spectrum (125 MHz, D_2O) of oxidised CH_3-α-L-rhamnopyranoside (m-Rhap) using 1.0 eq of periodate, showing the signals for product **2**: 14.6 (C_6); 55.0 (C_7); 64.7 (C_5); 69.3 (C_2); 88.5 (C_3); 91.0 (C_4); 96.0 (C_1) ppm.

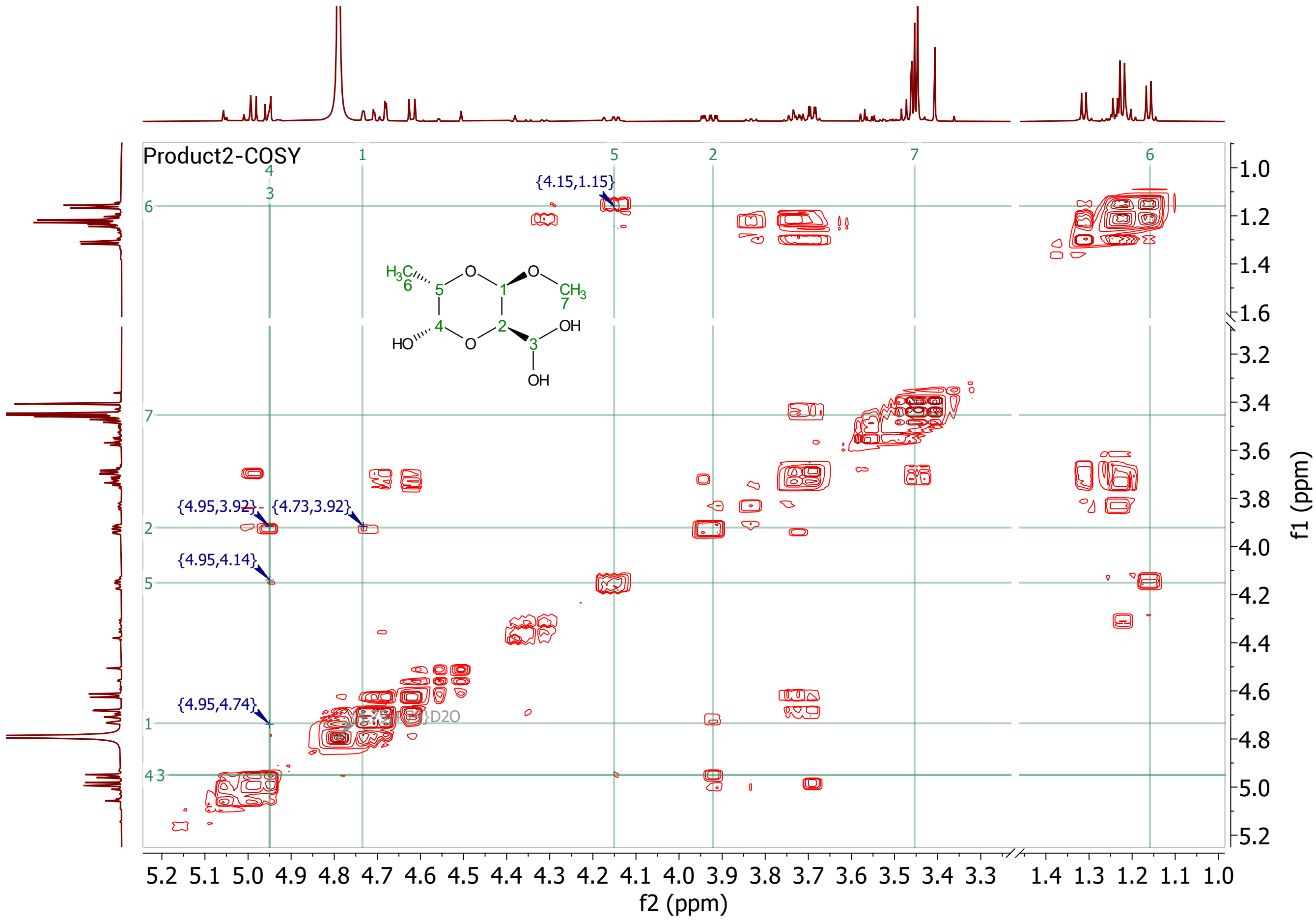

Figure S35: COSY spectrum (600 MHz, D_2O) of oxidised CH_3-α-L-rhamnopyranoside (m-Rhap) using 1.0 eq of periodate, showing the signals for product **2** with crosspeaks between H1 and H2 (4.73 and 3.92 ppm), H2 and H3 (3.92 and 4.95 ppm), H4 and H5 (4.95 and 4.14 ppm), and H5 and H6 (4.15 and 1.15 ppm).

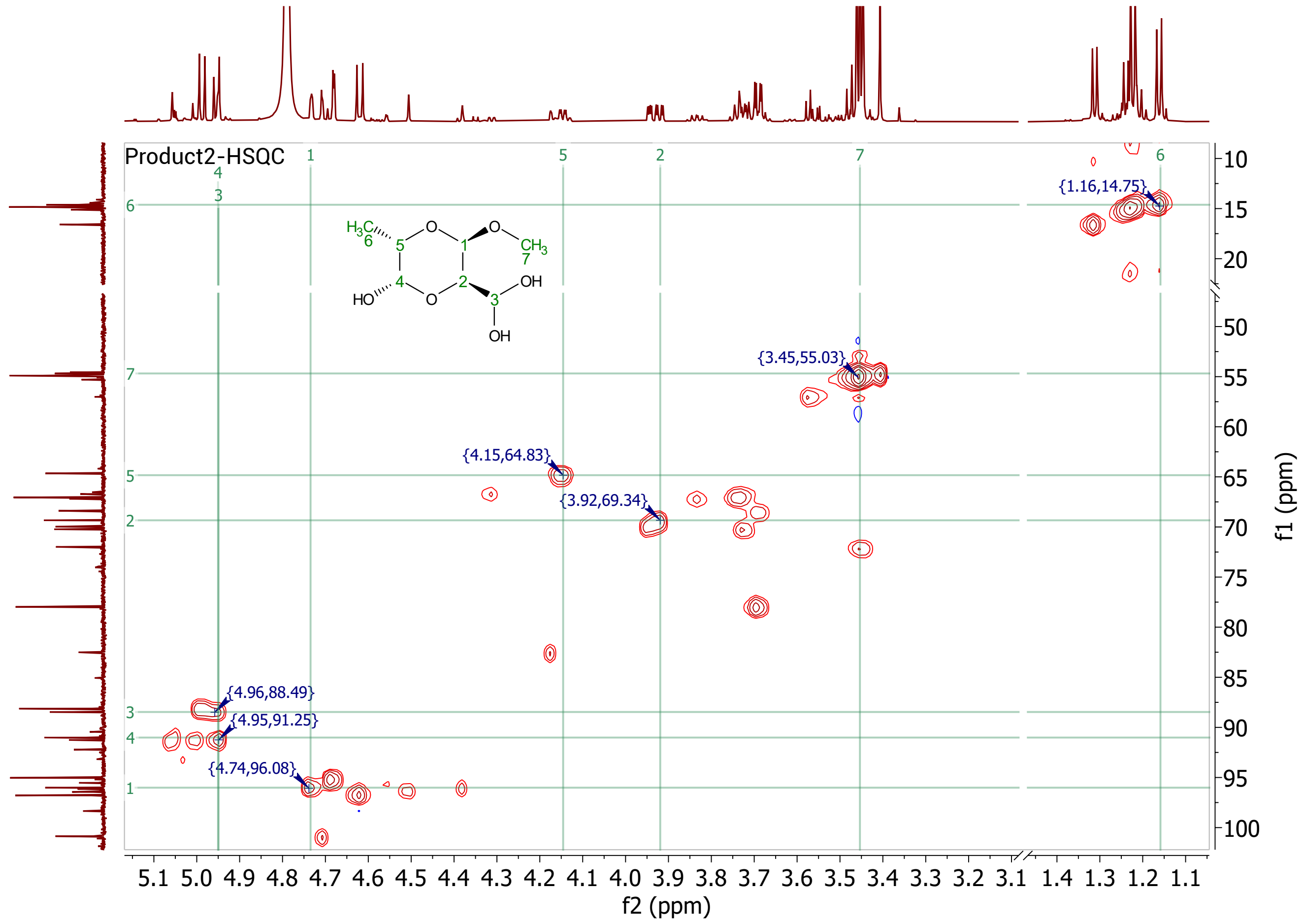

Figure S36: HSQC spectrum (600 MHz, D_2O) of oxidised CH_3-α-L-rhamnopyranoside (m-Rhap) using 1.0 eq of periodate, showing the signals for product **2** with crosspeaks between H1 and C1 (4.74 and 96.0 ppm), H2 and C2 (3.92 and 69.3 ppm), H3 and C3 (4.96 and 88.5 ppm), H4 and C4 (4.95 and 91.3 ppm), H5 and C5 (4.15 and 64.8 ppm), H6 and C6 (1.16 and 14.8 ppm), and H7 and C7 (3.45 and 55.0 ppm).

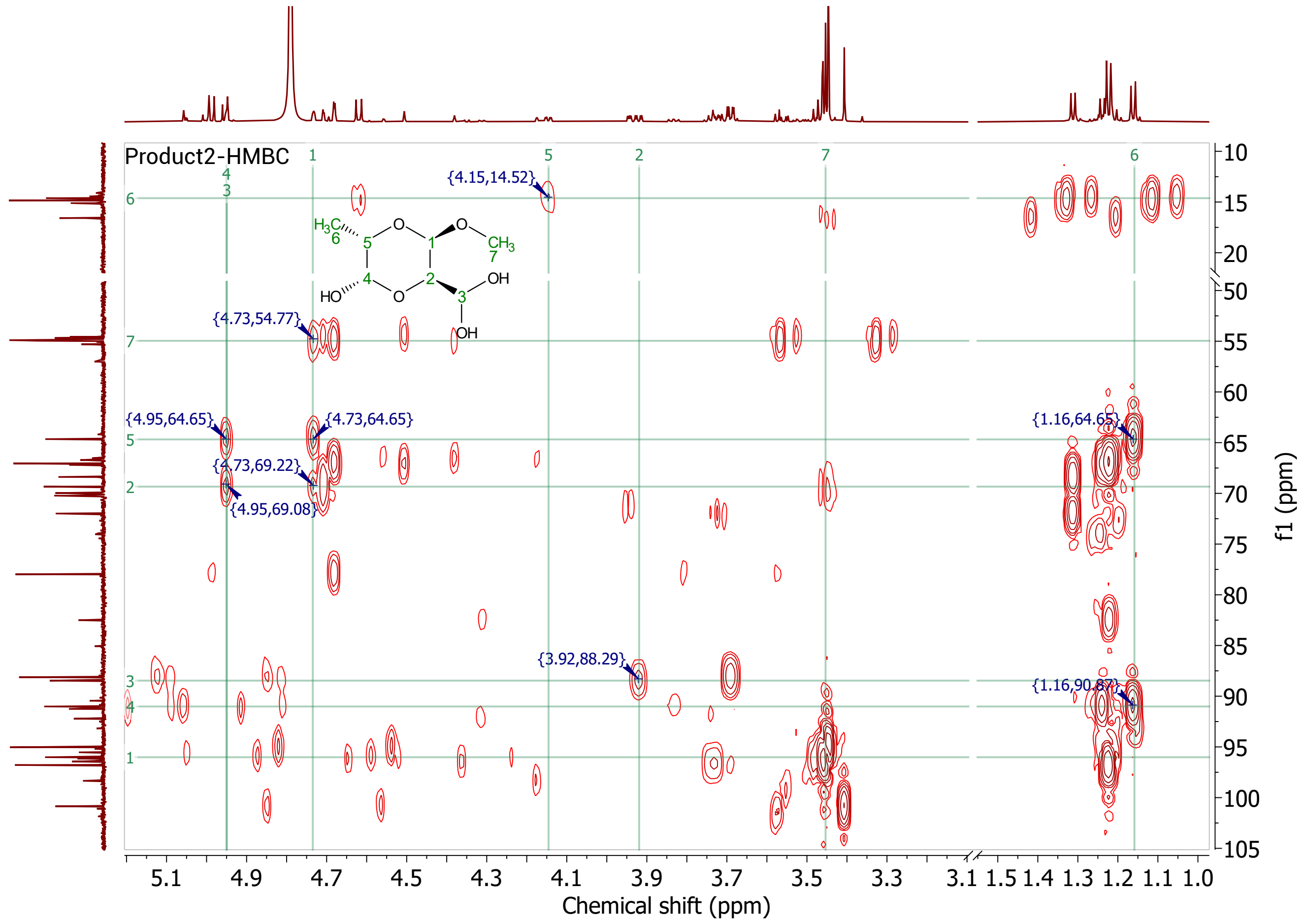

Figure S37: HMBC spectrum (600 MHz, D_2O) of oxidised CH_3-α-L-rhamnopyranoside (m-Rhap) using 1.0 eq of periodate, showing the signals for product **2**, with crosspeaks between H1 and C2 (4.73 and 69.2 ppm), H1 and C5 (4.73 and 64.7 ppm), H1 and C7 (4.73 and 54.8 ppm), H2 and C3 (3.92 and 88.3 ppm), H3 and C2 (4.95 and 69.1 ppm), H4 and C5 (4.95 and 64.7 ppm), H6 and C4 (1.16 and 90.9 ppm), and H6 and C5 (1.16 and 64.7 ppm).

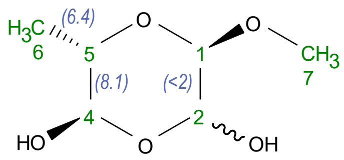

Figure S38: Structure of product **3** with between brackets the J-coupling values of H1, H2, H4, H5 and H6 as obtained from the ^{1}H-NMR spectrum (Fig. S40), which were used to determine the stereochemistry of product **3**. The not observable J-coupling between H1 and H2 indicates either equatorial-equatorial or axial-equatorial coupling.[13] With H1 pointing down, this means that H2 can point either up or down, and the same is the case for the hydroxyl group on the C2 position. The large J-coupling of 8.1 Hz between H4 and H5 indicates an axial-equatorial interaction,[13] and with H5 pointing up, this means that H4 has to be pointing up and the hydroxyl group on the C4 atom has to be pointing down. H3 or C3 are not present in product **3**, as they have formed formic acid, due to double oxidation (see Fig. S39).

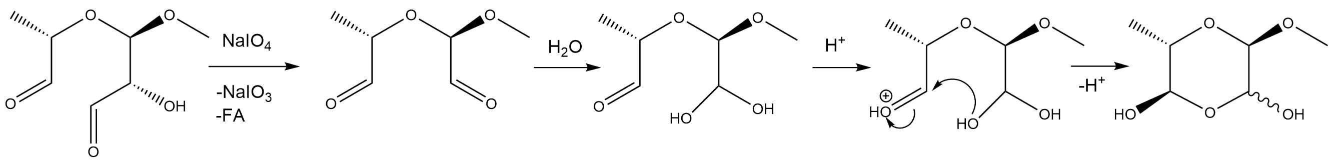

Figure S39: Proposed mechanism for the formation of product **3**. After the initial oxidation of either the syn or the anti diol, periodate oxidises once again, forming an aldehyde on the C2 and C4 positions. The C3 carbon is converted to formic acid. Hydration on the C2 position, followed by protonation of the aldehyde on the C4 position, allows for ring-closing and deprotonation to formation of a substituted dioxane, product **3**.

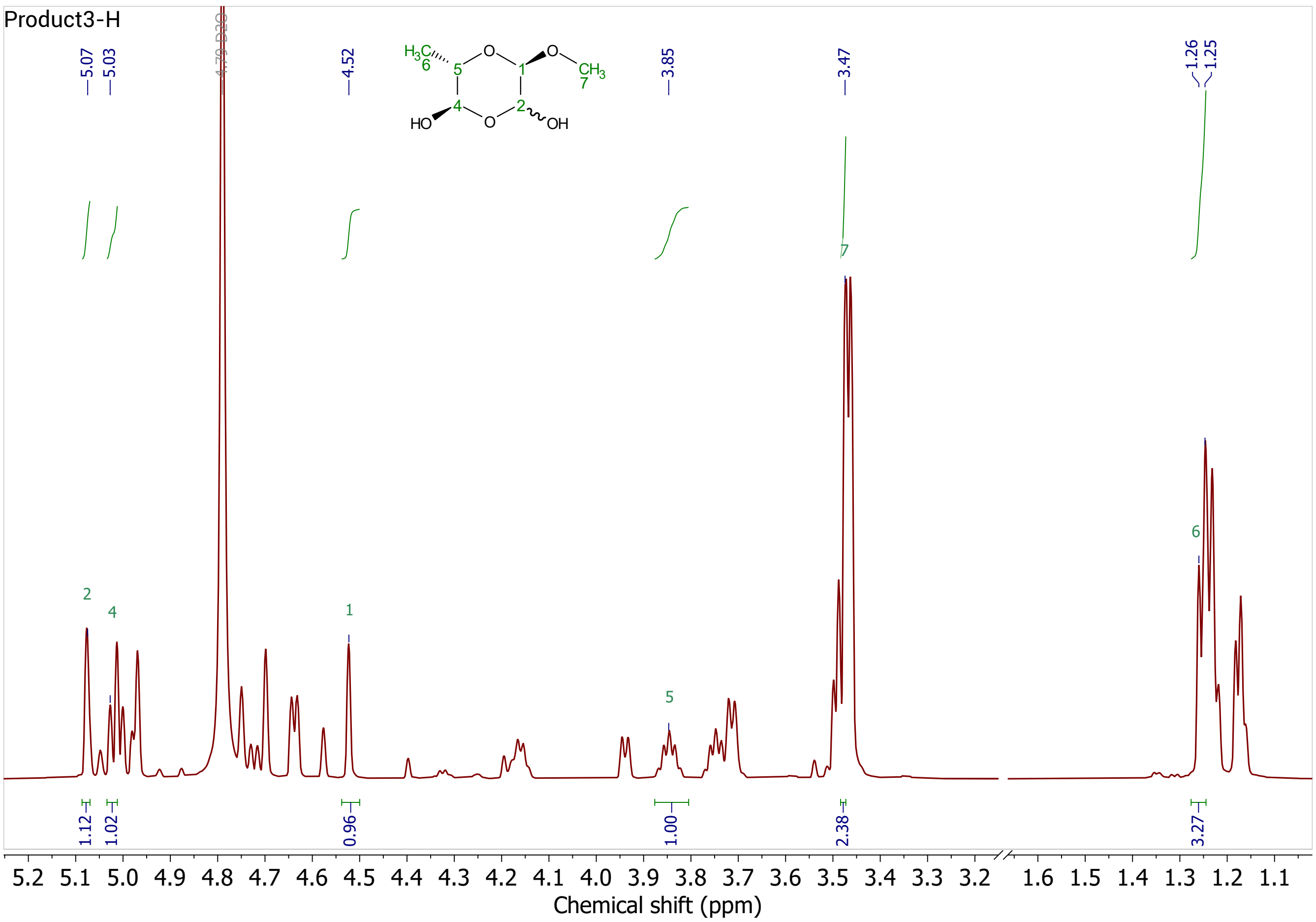

Figure S40: ^{1}H-NMR spectrum (600 MHz, D_2O) of oxidised CH_3-α-L-rhamnopyranoside (m-Rhap) using 2.0 eq of periodate, showing the signals for one of the two possible enantiomers of product **3**: 1.26 (d, J = 6.4 Hz, 3H, H6); 3.47 (s, 3H, H7); 3.85 (dd, J = 8.1, 6.4 Hz, 1H, H5); 4.52 (s, 1H, H1); 5.03 (d, J = 8.00 Hz, 1H, H4); 5.07 (s, 1H, H2).

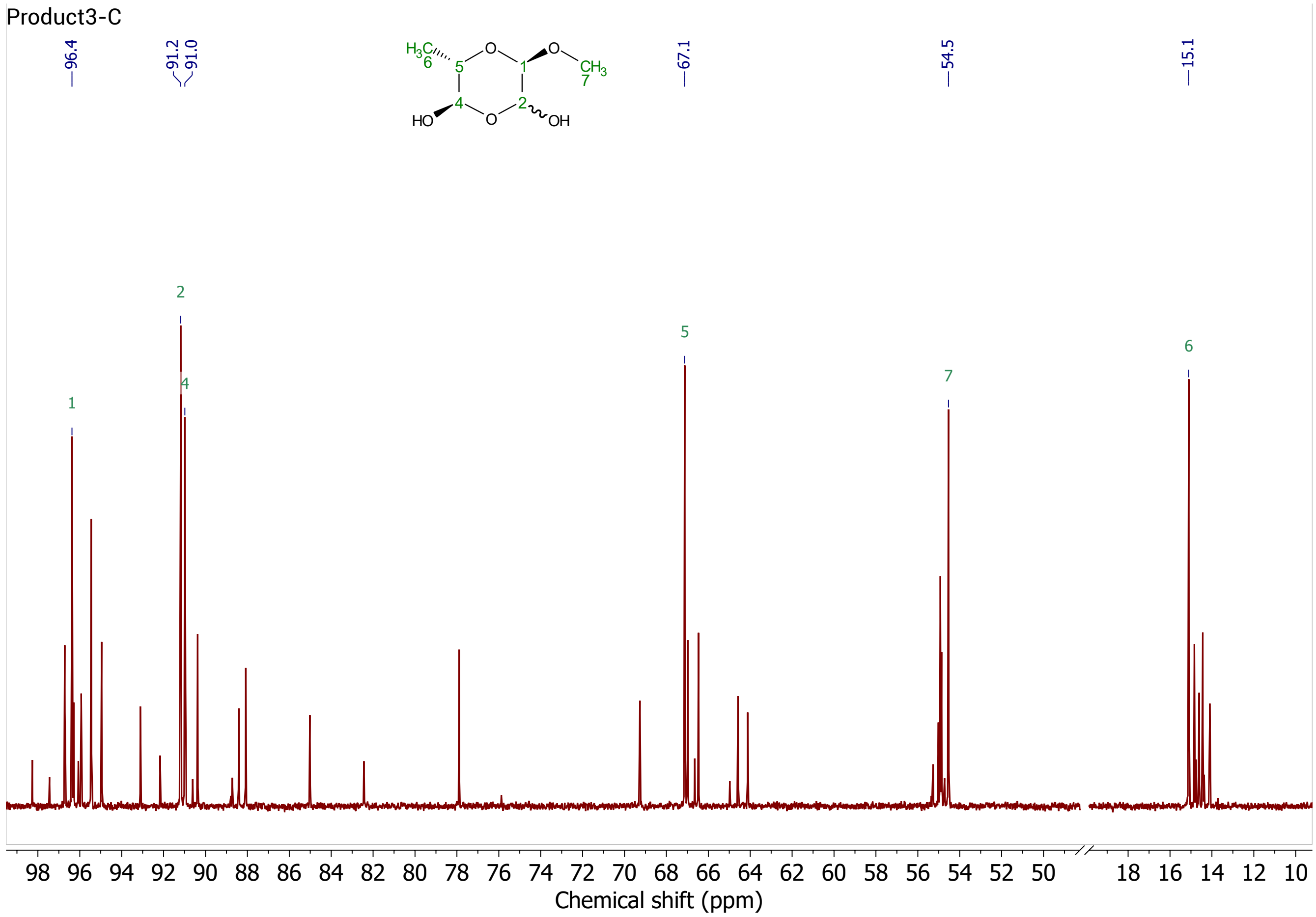

Figure S41: ^{13}C-NMR spectrum (125 MHz, D_2O) of oxidised CH_3-α-L-rhamnopyranoside (m-Rhap) using 2.0 eq of periodate, showing the signals for product **3**: 15.1 (C6); 54.5 (C7); 67.1 (C5); 91.0 (C4); 91.2 (C2); 96.4 (C1).

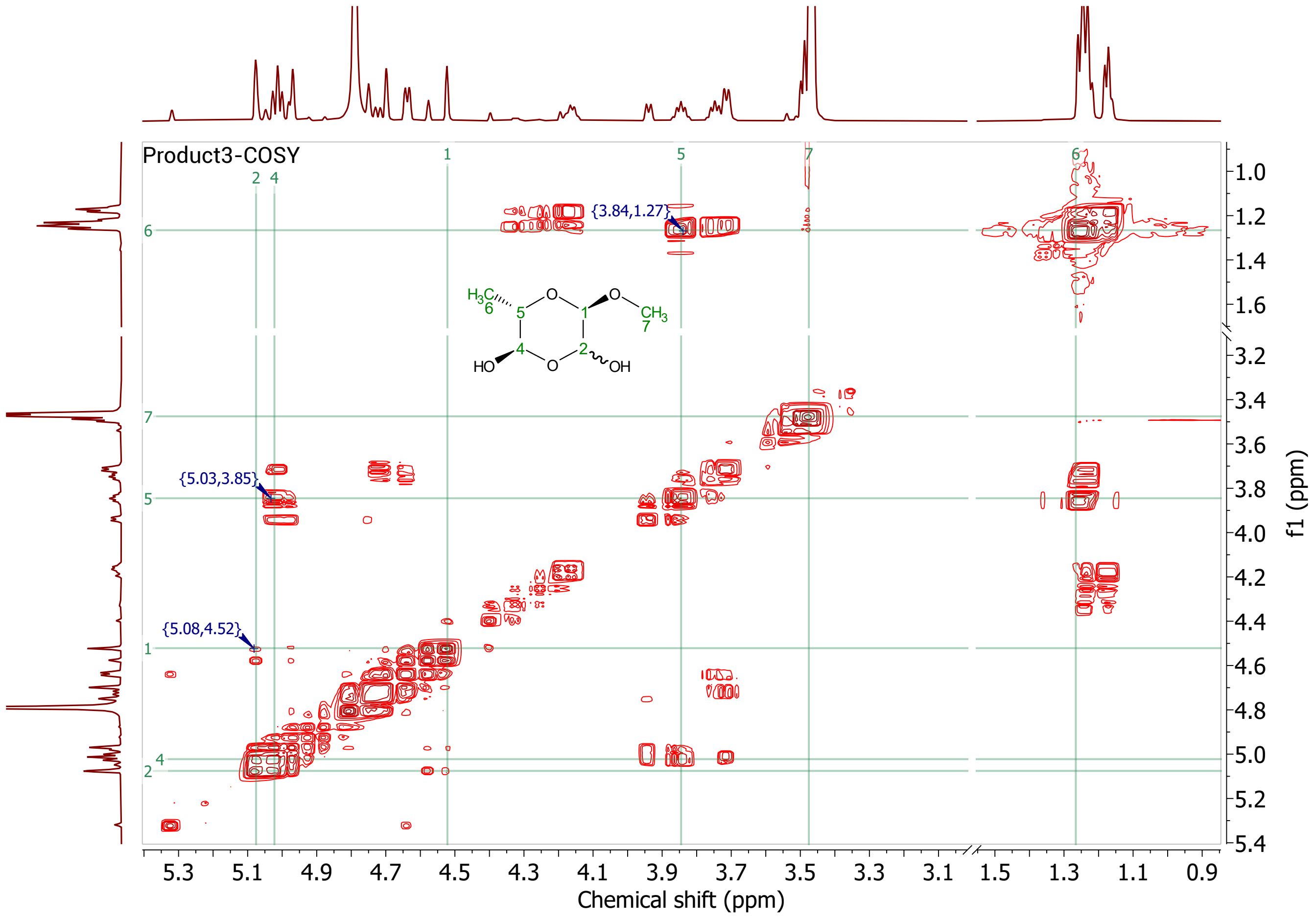

Figure S42: COSY spectrum (600 MHz, D_2O) of oxidised CH_3-α-L-rhamnopyranoside (m-Rhap) using 2.0 eq of periodate, showing the signals for product **3** with crosspeaks between H1 and H2 (4.52 and 5.08 ppm), H4 and H5 (5.08 and 4.52 ppm), and H5 and H6 (3.84 and 1.27 ppm).

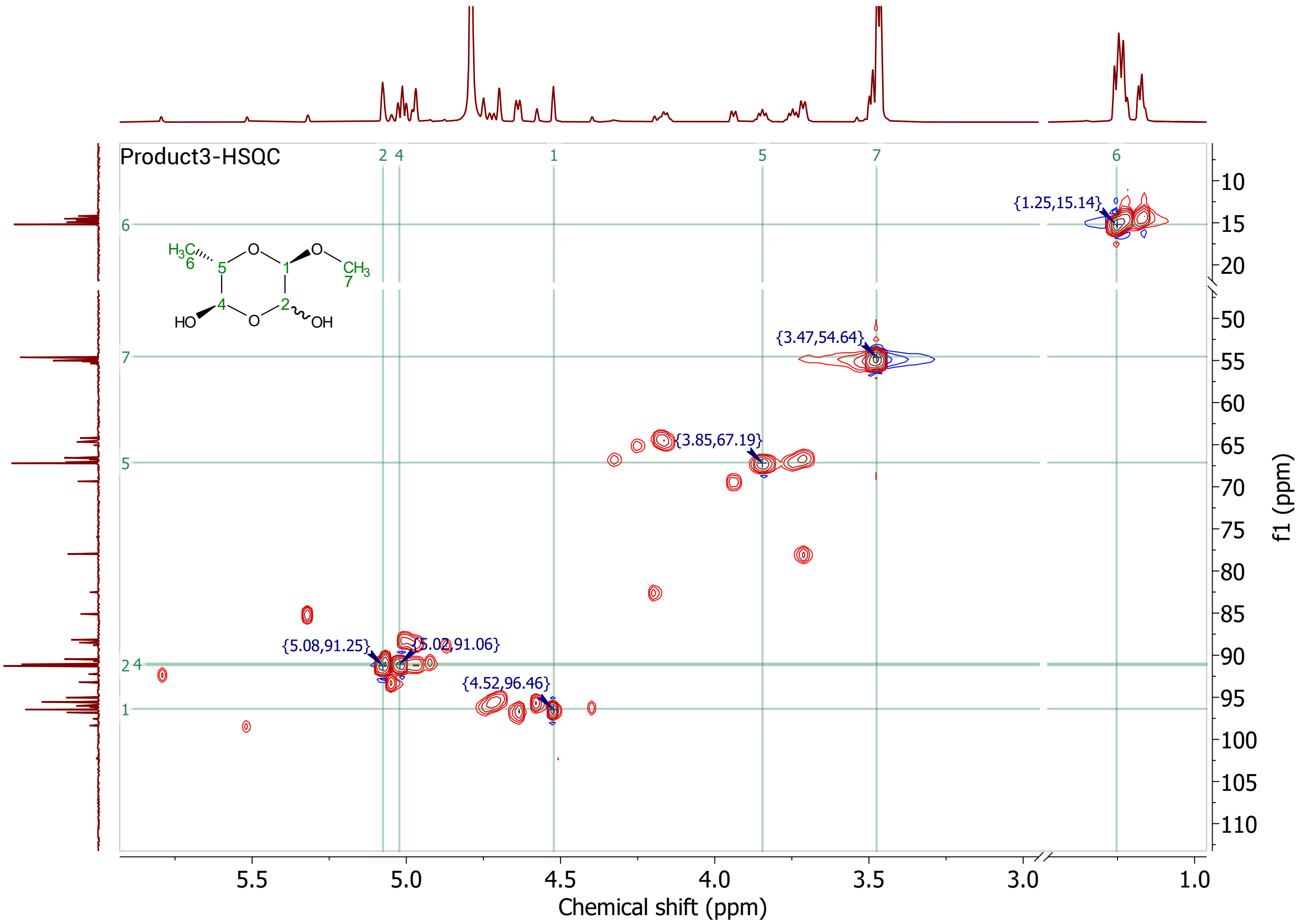

Figure S43: HSQC spectrum (600 MHz, D_2O) of oxidised CH_3-α-L-rhamnopyranoside (m-Rhap) using 2.0 eq of periodate, showing the signals for product **3** with crosspeaks between H1 and C1 (4.52 and 96.5 ppm), H2 and C2 (5.02 and 91.1 ppm), H4 and C4 (5.02 and 91.1 ppm), H5 and C5 (3.85 and 67.2 ppm), H6 and C6 (1.25 and 15.1 ppm), and H7 and C7 (3.47 and 54.6 ppm).

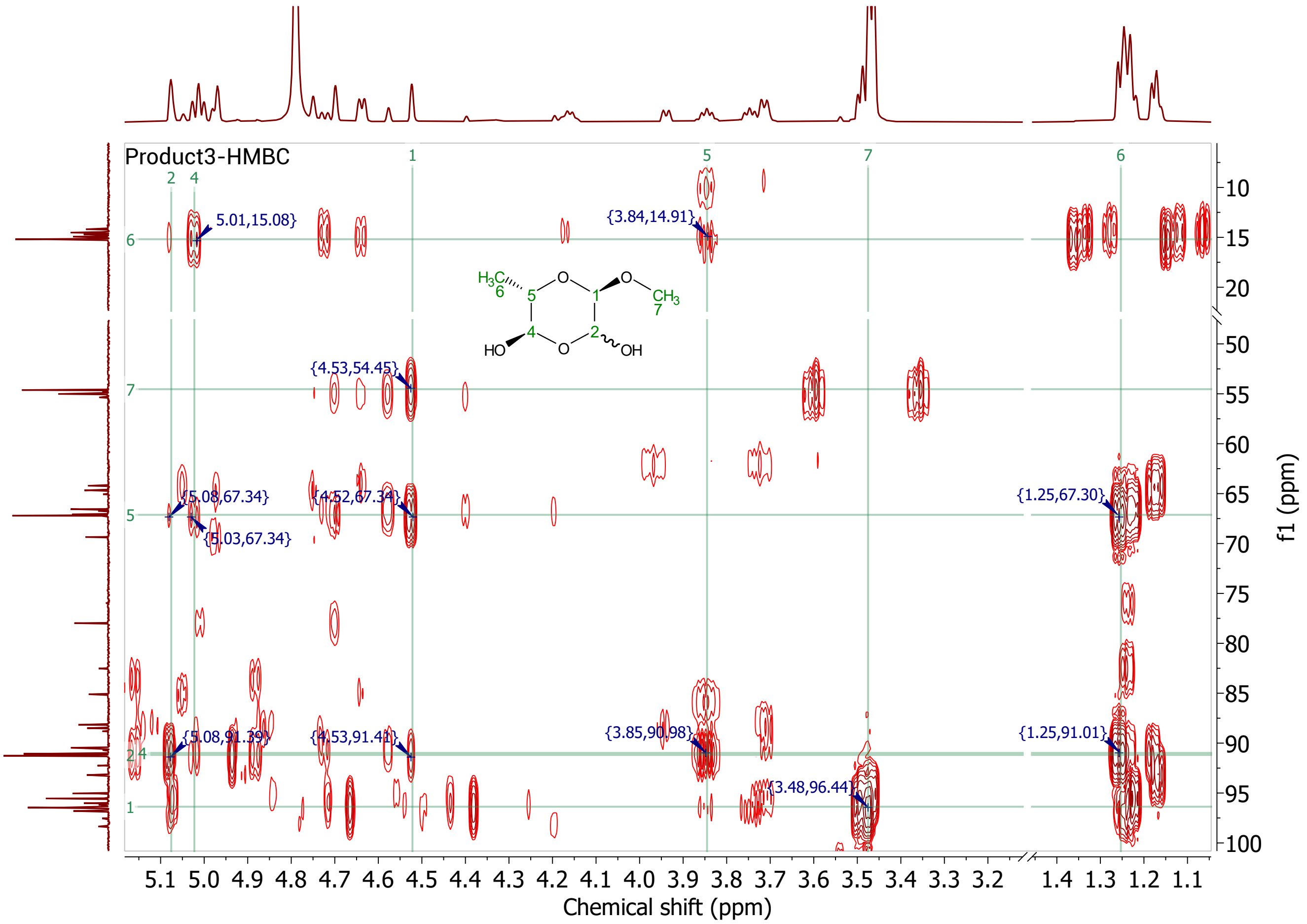

Figure S44: HMBC spectrum (600 MHz, D_2O) of oxidised CH_3-α-L-rhamnopyranoside (m-Rhap) using 2.0 eq of periodate, showing the signals for product **3** with crosspeaks between H1 and C2 (4.53 and 91.4 ppm), H1 and C5 (4.52 and 67.3 ppm), H1 and C7 (4.53 and 54.5 ppm), H2 and C4 (5.08 and 91.4 ppm), H2 and C5 (5.08 and 67.3 ppm), H4 and C5 (5.03 and 67.3 ppm), H4 and C6 (5.01 and 15.1 ppm), H6 and C4 (1.25 and 91.0 ppm), and H6 and C5 (1.25 and 67.3 ppm).

11. DFT calculations

Computational details - NMR

All density functional theory and NMR property calculations have been performed using ORCA 5.0.2.[14] Initial structures were drawn using Avogadro,[15] conformers were generated with meta-dynamics using CREST[16] with the GFN2-xTB method[17] and ALPB water solvation.

- part0: b97-d3/def2-SV(P) // GFNn-xTB (Input geometry) (threshold = 4.0 kcal/mol)
- part1: r2scan-3c + SMD[h2o] + GmRRHO(GFN2[alpb]-bhess) // GFNn-xTB (Input geometry) (threshold = 3.5 kcal/mol)
- part2: r2scan-3c + SMD[h2o] + GmRRHO(GFN2[alpb]-bhess) // r2scan-3c[SMD] (threshold = 2.5 kcal/mol)
- NMR shift: pbe0-d4/pcsseg-3 + SMD[h2o]
- NMR coupling: pbe0-d4/pcJ-3 + SMD[h2o]

Briefly, the results from crest are taken and prescreened using b97-d3/def2-SV(P) and later r2scan-3c with a cheap GmRRHO free energy correction before they are optimized using r2scan-3c[SMD]. Between each step, conformers higher in energy than the threshold are discarded. Finally the NMR shifts are calculated using pbe0-d4/pcsseg-3[18,19] and coupling constants using pbe0-d4/pcJ-3,[20] both with SMD water solvation. From the NMR shifts and couplings the spectra are calculated and the conformers are weighted using ANMR.[21]

For the reference, TMS was optimized and calculated using the same methods as above and its chemical shift was subtracted from the calculated values.

Computational details - energies

The Gibbs free energies were obtained for the conformers at the end of step 2 at the r2scan-3c + SMD[h2o] + GmRRHO(GFN2[alpb]-bhess) // r2scan-3c[SMD] level. Energies were weighted according to:

$$< E >= \sum_i E_i P_i$$

With:

$$P_i = \frac{\exp\left(\frac{-E_i}{k_B T}\right)}{\sum_j \exp\left(\frac{-E_j}{k_B T}\right)}$$

Structure	Gibbs energy (kcal/mol)	Relative energy (kcal/mol)
**1**	-455714.9327	0
**2**	-455714.6716	0.261089
**4**	-455710.744	4.18872
**5**	-455712.4878	2.44489
**3**	-383866.8391	0.194027
**6**	-383866.6675	0.365611
**7**	-383866.6098	0.423306
**8**	-383867.0331	0
m-Rhap	-408515.0251	

Table S2: Calculated energies for the various products that can be formed upon the oxidation of m-Rhap by periodate.

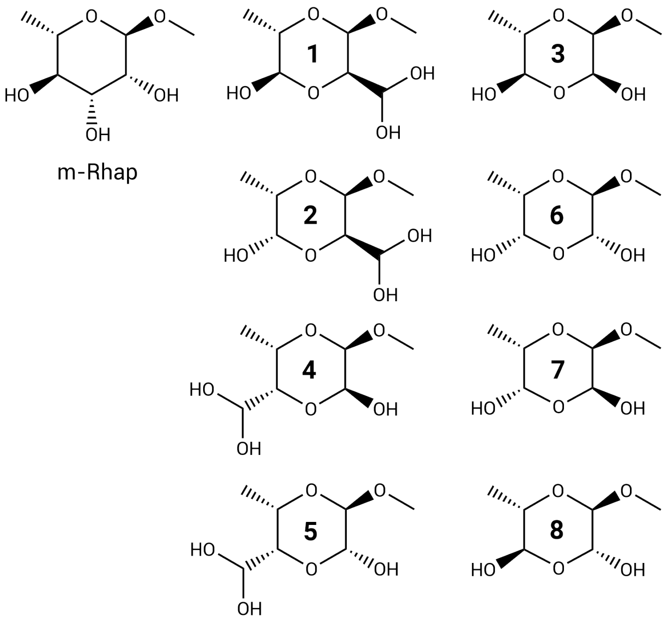

Figure S45: Structures of possible products from the oxidation of m-Rhap by periodate used for the DFT calculations. Product **1**, **2**, and product **3** or its enantiomer **8** were found experimentally.

Calculated spectra

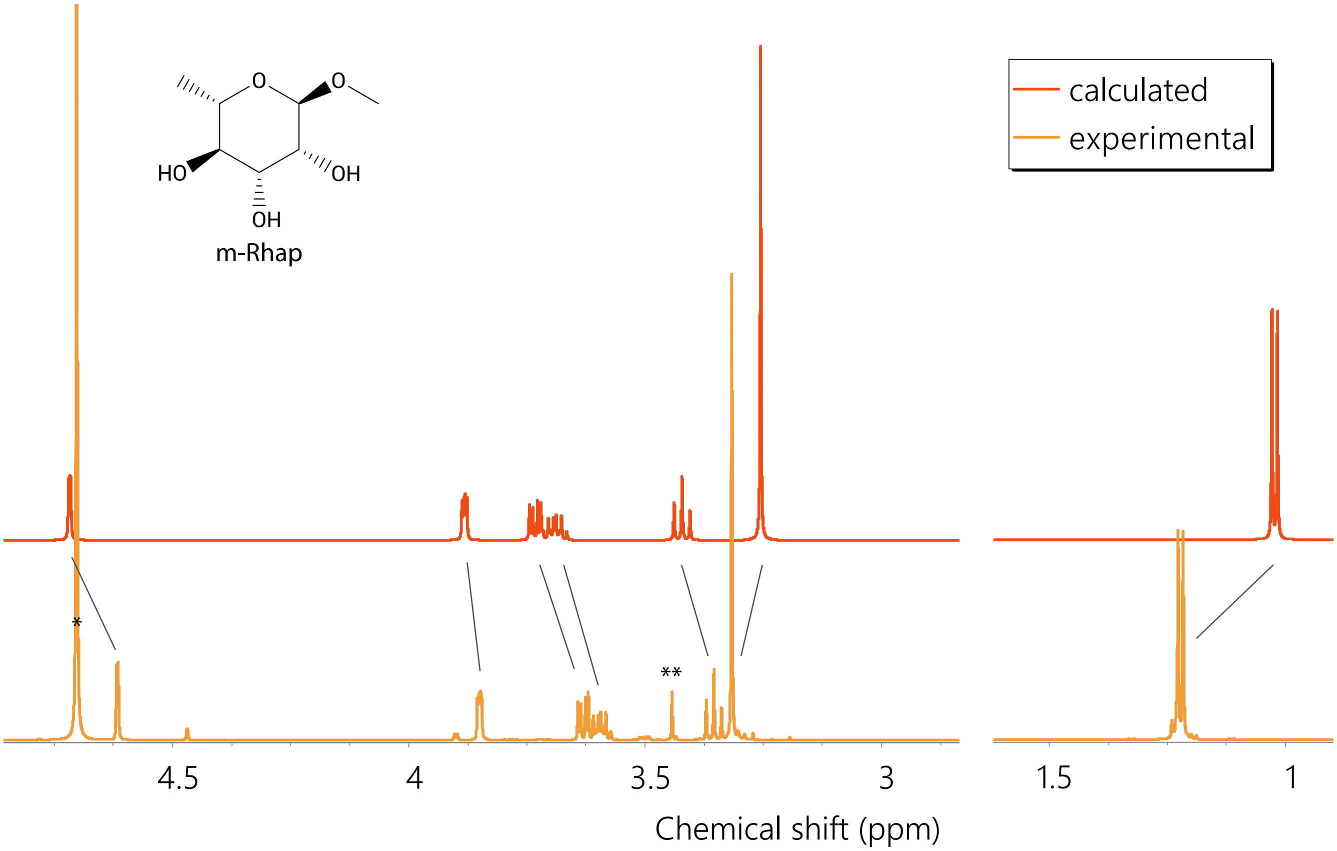

Figure S46: The (top) calculated and (bottom) experimental ^{1}H-NMR spectra of m-Rhap in D_2O showing the correspondence in signal chemical shifts and coupling patterns. * is HDO and ** is remaining methanol.

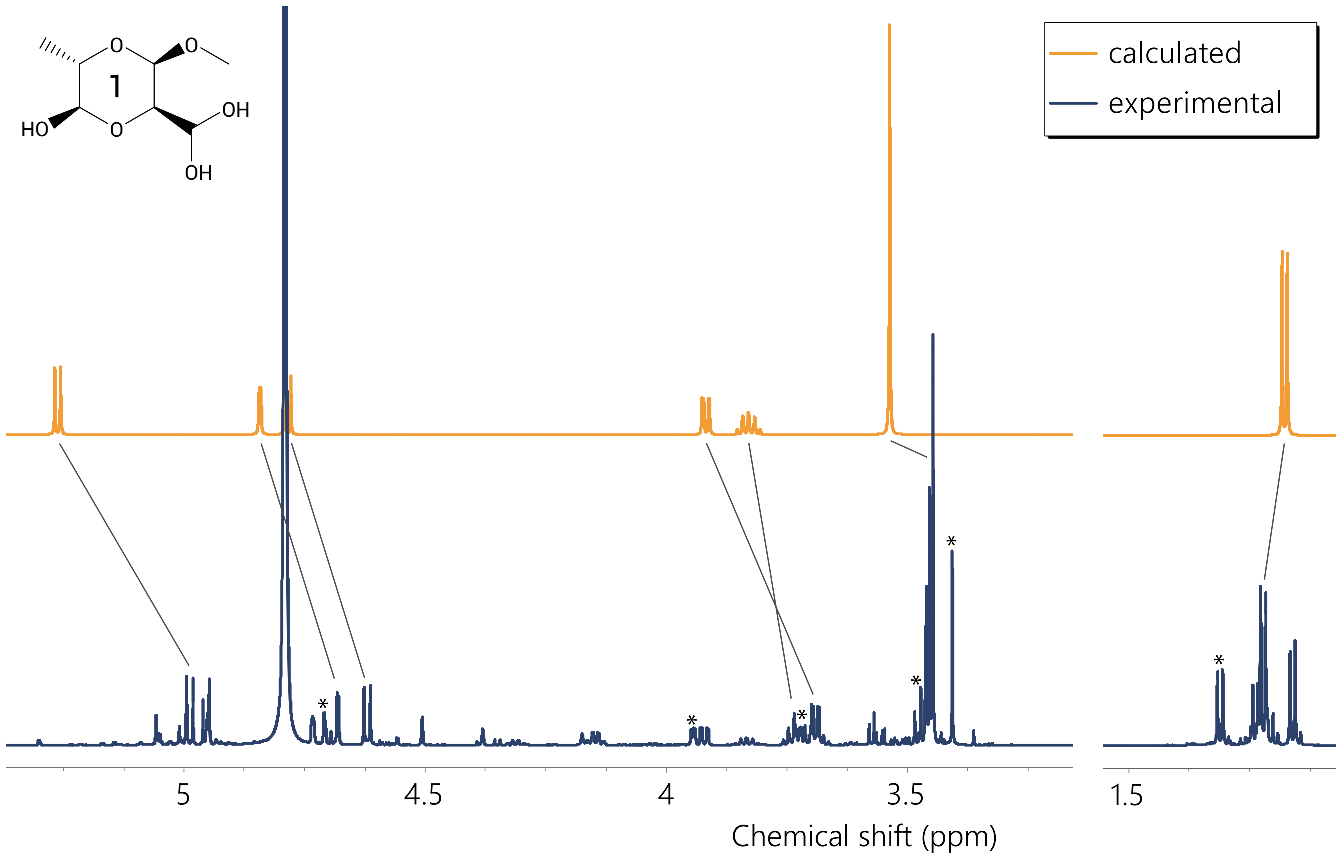

Figure S47: The (top) calculated spectrum of product **1** and (bottom) the signals of product **1** in the experimental ^{1}H-NMR spectrum of the mixture of products obtained for the oxidation of m-Rhap with periodate D_2O. Signals marked with * are from the m-Rhap starting material.

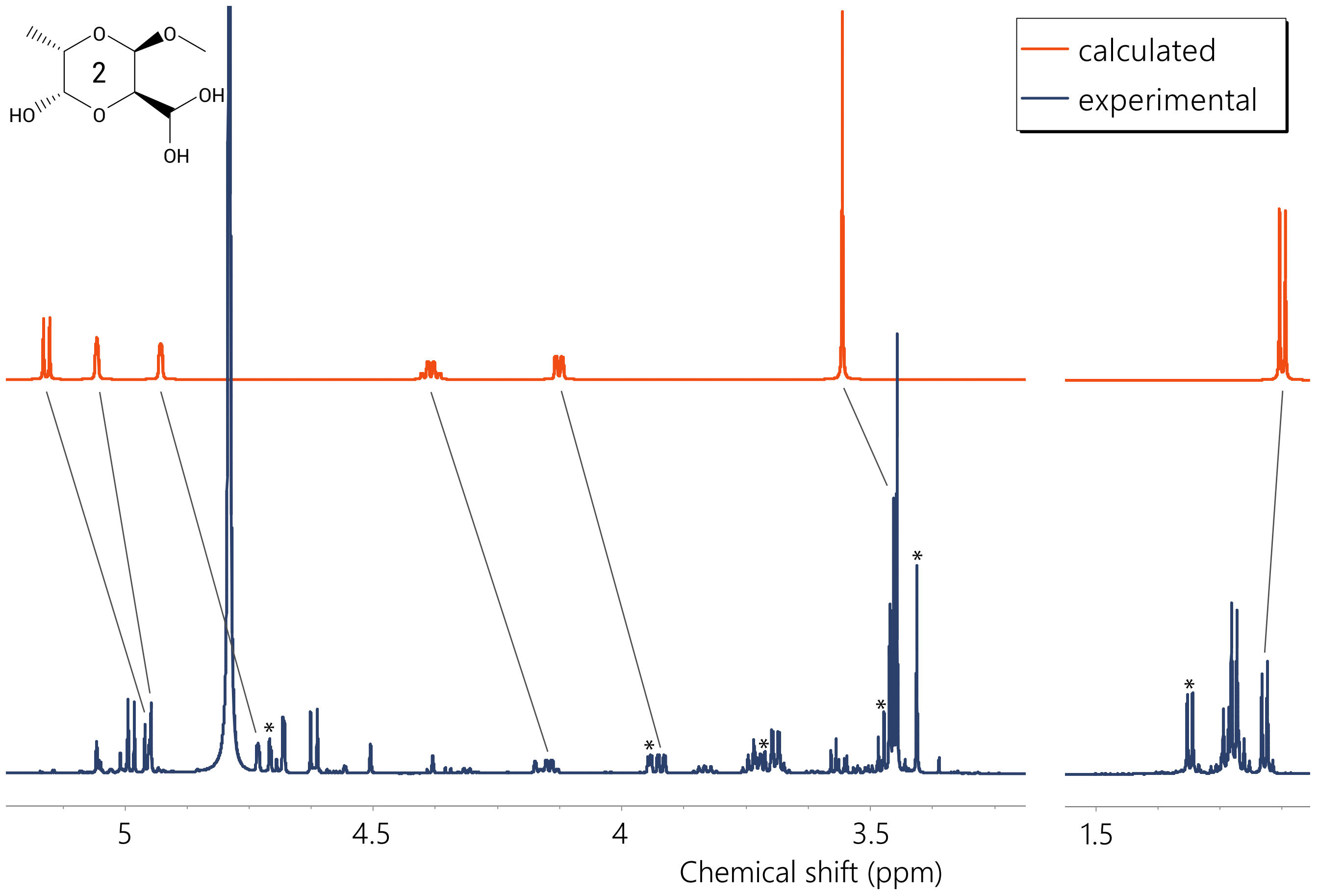

Figure S48: The (top) calculated spectrum of product **2** and (bottom) the signals of product **2** in the experimental ^{1}H-NMR spectrum of the mixture of products obtained for the oxidation of m-Rhap with periodate D_2O. Signals marked with * are from the m-Rhap starting material.

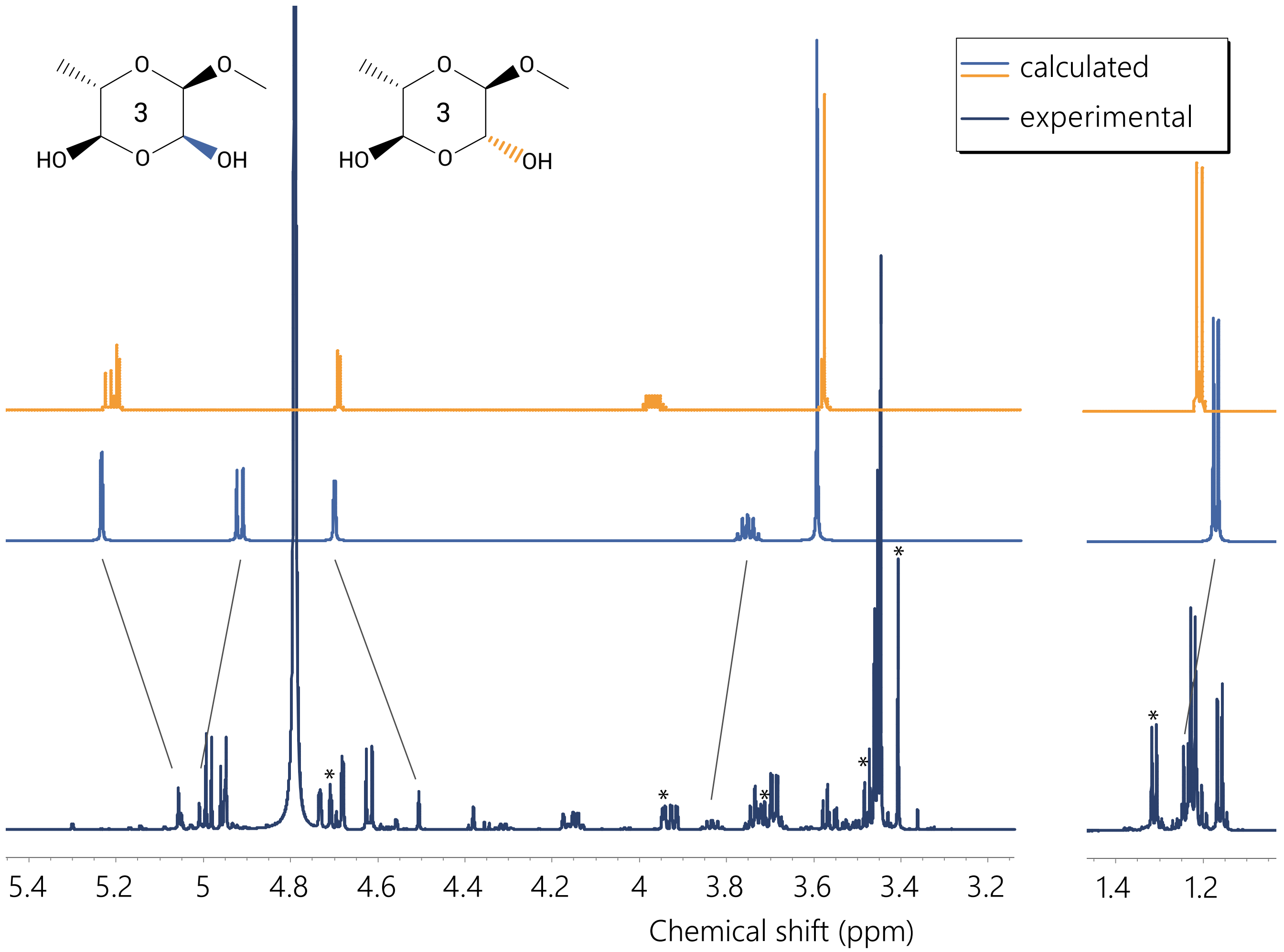

Figure S49: The (top) calculated spectra of the two possible enantiomers of product **3** and (bottom) the signals of product **3** in the experimental ^{1}H-NMR spectrum of the mixture of products obtained for the oxidation of m-Rhap with periodate D_2O. Signals marked with * are from the m-Rhap starting material.

12. Solid state ^{13}C NMR spectroscopy

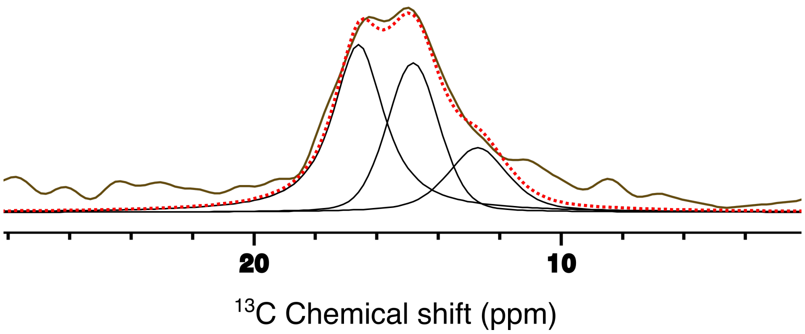

Figure S50: The ^{13}C solid state NMR spectrum of oxidised GA1, showing the deconvolution of the signal for the methyl carbon of rhamnopyranoside units in the sample isolated by precipitation in ethanol.

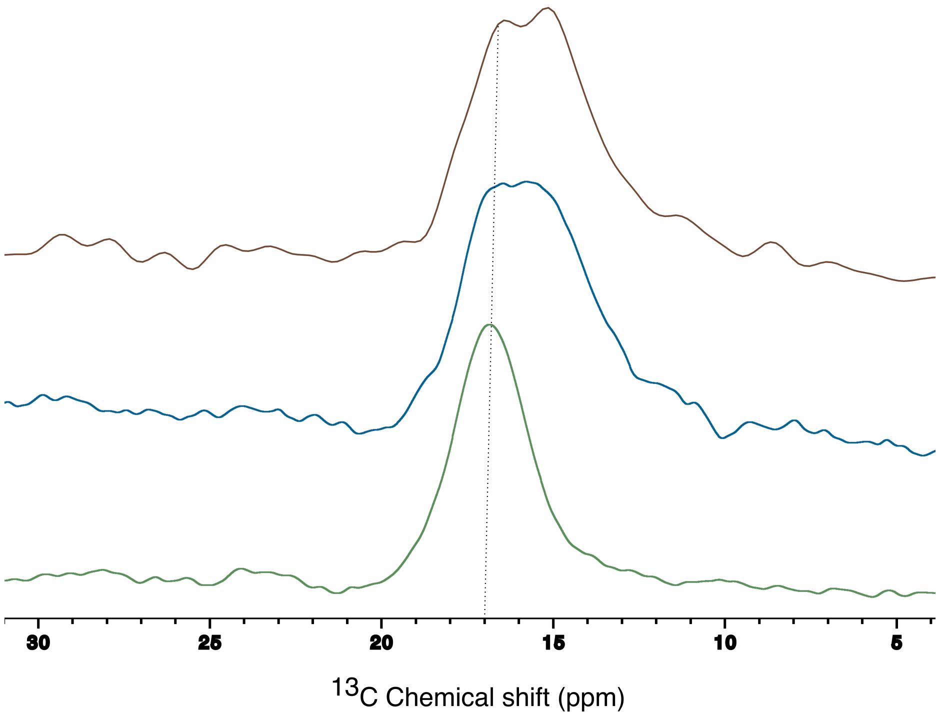

Figure S51: The ^{13}C solid state NMR spectrum of oxidised GA1, showing rhamnopyranoside methyl signal(s) in unoxidised GA1 (green), oxGA1 isolated by freeze drying (blue) and oxGA1 isolated by precipitation in ethanol (red).

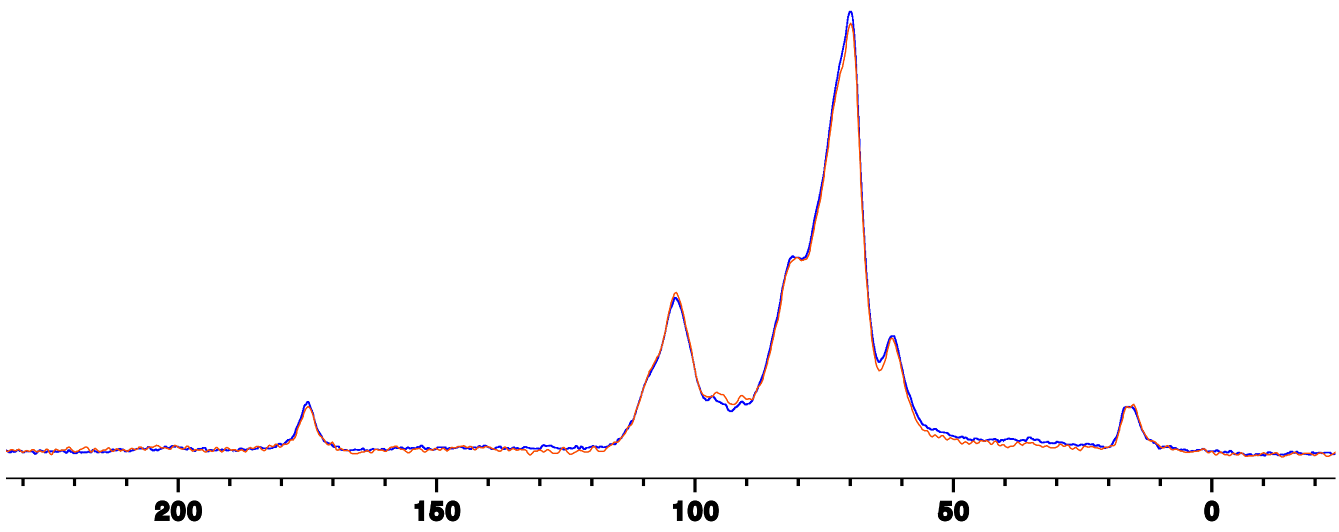

Figure S52: The ^{13}C solid state NMR spectra of oxidised GA1, isolated by freezedrying (blue) and precipitation in ethanol (orange).

12. References

[1] S. P. Nie, C. Wang, S. W. Cui, Q. Wang, M. Y. Xie and G. O. Phillips, *Food Hydrocoll.*, 2013, **31**, 42–48.

[2] R. Darvishi, H. Moghadas and A. Moshkriz, *Korean J. Chem. Eng.*, 2022, **39**, 1350–1360.

[3] J. Stefanovic, D. Jakovljevic, G. Gojgic-Cvijovic, M. Lazic and M. Vrvic, *J. Appl. Polym. Sci.*, 2013, **127**, 4736–4743.

[4] T. G. Halsall, E. L. Hirst and J. K. N. Jones, *J. Chem. Soc. (Res.)*, 1947, 1427.

[5] K. K. Nishi and A. Jayakrishnan, *Biomacromolecules*, 2004, **5**, 1489–1495.

[6] J. M. Bobbitt, *Adv. Carbohydr. Chem.*, 1956, **11**, 1–41.

[7] J. A. Sirviö, H. Liimatainen, J. Niinimäki and O. Hormi, *RSC Adv.*, 2013, **3**, 16590–16596.

[8] M. Siller, H. Amer, M. Bacher, W. Roggenstein, T. Rosenau and A. Potthast, *Cellulose*, 2015, **22**, 2245–2261.

[9] S. Vicini, E. Princi, G. Luciano, E. Franceschi, E. Pedemonte, D. Oldak, H. Kaczmarek and A. Sionkowska, *Thermochim. Acta*, 2004, **418**, 123–130.

[10] F. Mecozzi, J. J. Dong, D. Angelone, W. R. Browne and N. N. Eisink, *Eur. J. Org. Chem.*, 2019, **2019**, 7151–7158.

[11] B. H. Nicolet and L. A. Shinn, *J. Am. Chem. Soc.*, 1939, **61**, 1615–1615.

[12] A. de Bruyn, M. Anteunis, R. de Gussem and G. G. Dutton, *Carbohydr. Res.*, 1976, **47**, 158–163.

[13] M. U. Roslund, P. Tähtinen, M. Niemitz and R. Sjöholm, *Carbohydr. Res.*, 2008, **343**, 101–112.

[14] F. Neese, *Wiley Interdiscip. Rev. Comput. Mol. Sci.*, 2012, **2**, 73–78.

[15] M. D. Hanwell, D. E. Curtis, D. C. Lonie, T. Vandermeersch, E. Zurek and G. R. Hutchison, *J. Cheminform.*, 2012, **4**, 1–17.

[16] P. Pracht and S. Grimme, *Chem. Sci.*, 2021, **12**, 6551–6568.

[17] C. Bannwarth, S. Ehlert, S. Grimme and B. P. S.-C. Tight-Binding, *J. Chem. Theory Comput.*, 2019, **15**, 1652–1671.

[18] C. Adamo and V. Barone, *J. Chem. Phys.*, 1999, **110**, 6158–6170.

[19] F. Jensen, *J. Chem. Theory Comput.*, 2015, **11**, 132–138.

[20] F. Jensen, *J. Chem. Theory Comput.*, 2006, **2**, 1360–1369.

[21] S. Grimme, F. Bohle, A. Hansen, P. Pracht, S. Spicher and M. Stahn, *J. Phys. Chem. A.*, 2021, **125**, 4039–4054.
